# Novel NAC Transcription Factor TaNAC67 Confers Enhanced Multi-Abiotic Stress Tolerances in *Arabidopsis*

**DOI:** 10.1371/journal.pone.0084359

**Published:** 2014-01-10

**Authors:** Xinguo Mao, Shuangshuang Chen, Ang Li, Chaochao Zhai, Ruilian Jing

**Affiliations:** The Key Laboratory for Crop Gene Resources and Germplasm Enhancement, Ministry of Agriculture, The National Key Facility for Crop Gene Resources and Genetic Improvement, Institute of Crop Science, Chinese Academy of Agricultural Sciences, Beijing, China; Cankiri Karatekin University, Turkey

## Abstract

Abiotic stresses are major environmental factors that affect agricultural productivity worldwide. NAC transcription factors play pivotal roles in abiotic stress signaling in plants. As a staple crop, wheat production is severely constrained by abiotic stresses whereas only a few NAC transcription factors have been characterized functionally. To promote the application of NAC genes in wheat improvement by biotechnology, a novel NAC gene designated *TaNAC67* was characterized in common wheat. To determine its role, transgenic *Arabidopsis* overexpressing *TaNAC67-GFP* controlled by the CaMV-35S promoter was generated and subjected to various abiotic stresses for morphological and physiological assays. Gene expression showed that *TaNAC67* was involved in response to drought, salt, cold and ABA treatments. Localization assays revealed that TaNAC67 localized in the nucleus. Morphological analysis indicated the transgenics had enhanced tolerances to drought, salt and freezing stresses, simultaneously supported by enhanced expression of multiple abiotic stress responsive genes and improved physiological traits, including strengthened cell membrane stability, retention of higher chlorophyll contents and Na^+^ efflux rates, improved photosynthetic potential, and enhanced water retention capability. Overexpression of *TaNAC67* resulted in pronounced enhanced tolerances to drought, salt and freezing stresses, therefore it has potential for utilization in transgenic breeding to improve abiotic stress tolerance in crops.

## Introduction

Crop plants constantly encounter unfavorable environmental conditions, including drought, high salinity, and extreme temperatures, that significantly affect both biomass and grain yield worldwide. It is well established that transcription factors (TFs) play essential roles in response to different abiotic stimuli [1]. TFs have been grouped into diverse families based on the conserved structural domains involved in DNA binding to *cis*-elements in promoters of target genes, or other functional modular structures. Recent accumulated evidence demonstrates that numerous TFs, such as DREB, bZIP, zinc-finger, MYB, WRKY and NAC, directly or indirectly regulate plant defense and stress responses [2]–[9].

The NAC (NAM, ATAF, and CUC) superfamily is one of the largest plant-specific TF families. Proteins in this family are characterized by a highly conserved DNA binding NAC domain in the N-terminal and a highly diversified transcriptional activation domain in the C-terminal [10]. More than one hundred NAC members have been identified in *Arabidopsis*, rice, soybean and *Populus trichocarpa* [1], [11]–[13]. Considerable evidence showing that NACs play important roles in plant development, including pattern formation of embryos and flowers [14], secondary wall formation [15]–[17], leaf senescence [18]–[20], and root development [3], [21]. NACs are also involved in responses to various of biotic and abiotic stresses, including disease, drought, salt, cold and low-oxygen stresses, and act as targets of miRNAs [22]. It is believed that NAC TFs have potential to improve biotic and abiotic stress tolerances in plants [23]–[25], and some examples are listed below.

In *Arabidopsis*, three NAC members, AtNAC019, AtNAC055 and AtNAC072 bind to the ERD1 promoter region and enhance tolerance to drought stress [26]. *AtNAC2* is involved in response to various plant hormones and participates in salt stress response and lateral root development [27]. ATAF1 and ATAF2, along with a counterpart HvNAC6 in barley, play important roles in response to drought and pathogen stresses [28]–[30]. Furthermore, AtNAC102 is involved in regulating seed germination under low-oxygen conditions [31]. In rice, several NAC TFs have been well characterized. Overexpression of *SNAC1/OsNAC1*, *OsNAC5*, *SNAC2/OsNAC6*, *OsNAC045*, *OsNAC052* and *OsNAC063* enhance tolerance to drought or multiple abiotic stresses [32]–[38]. In soybean, *GmNAC11* and *GmNAC20* respond differentially to abiotic stresses and phytohormones. Overexpression of *GmNAC20* enhances salt and freezing tolerances, however, *GmNAC11* overexpression only improves salt tolerance in transgenic *Arabidopsis* [21]. In cotton, six NAC members were cloned and their differential expression patterns induced by abiotic stresses and ABA were characterized [39]. In wheat, multiple NAC genes have been isolated, and different NACs show diverse expression patterns or play various roles in response to environmental stimuli. TaGRAB1 and TaGRAB2 are involved in interaction with the Rep A protein of a geminivirus and thus inhibit DNA replication of wheat dwarf geminivirus in wheat cells [40]. *TaNAC69-1*, *TtNAMB-2*, *TaNAC4* and *TaNAC8* participate in response to multi-abiotic or/and biotic stresses [41]–[43]. Overexpression of *TaNAC2*, *TaNAC2a*, and *TaNAC69* improve tolerances to abiotic stresses in transgenic plants [3], [44]–[46].

Production of the world wheat crop is severely constrained by abiotic stresses, especially by drought. Only a few wheat NAC TFs have been well characterized functionally. To promote the application of NAC genes in wheat improvement by biotechnology, we characterized the expression patterns of *TaNAC67* in different tissues, and identified its dynamic responses to water deficit, high salinity, low temperature and ABA treatments. Transgenic experiments showed that overexpression of *TaNAC67* in *Arabidopsis* significantly enhanced tolerances to drought, salt and freezing stresses. Morphological assays revealed that overexpression of *TaNAC67* had no negative effects on the growth of transgenic plants, thus it has potential to improve abiotic tolerance in plants.

## Materials and Methods

### Plant materials and water-stress experiments

Wheat (*Trticum aestivum* L.) genotype “Hanxuan 10” with a significant degree of drought tolerance was used in this study. Growth conditions were as previously described [47]. Two-leaf seedlings were treated with polyethylene glycol-6000 (PEG-6000, −0.5 MPa) solution, 250 mM NaCl, low temperature (4°C), and 50 µM ABA. The treated plants were stressed in the PEG-6000 and NaCl solutions, sprayed with ABA, or cultured in low temperature conditions for 1, 3, 6, 12, 24, 48, and 72 h.

To investigate the genomic origin of the target gene, 20 accessions of various wheat species, including four A genome (*T*. *urartu*) accessions, four S genome (*Aegilops speltoides*) accessions, four D genome (*Ae*. *tauschii*) accessions, four accessions with the AB genome (three *T. dicoccoides* and one *T. dicoccum*), and four hexaploid wheat accessions were selected to perform PCR (Table S1 in File S1). Forty one nulli-tetrasomic lines of Chinese Spring (CS) were used to identify the chromosomal locations of the target gene.

*Arabidopsis thaliana* (Columbia ecotype), chosen for transgenic analysis, was grown in a controlled environment chamber at 22°C, with a 12 h/12 h photoperiod, a light intensity of 120 µmol m^−2^ s^−1^ and 70% relative humidity.

### Isolation of the full-length cDNA and sequence analysis of *TaNAC67*

A 450 bp cDNA fragment, encoding a NAC-like N-terminal domain obtained by sequencing from wheat suppression subtractive cDNA libraries treated with PEG-6000 [48], was used as a query probe to screen a wheat full-length cDNA library constructed with different tissues by the optimized Cap-trapper method [49]. Four candidate clones were obtained by nucleotide blast (blastn), and the full-length cDNA of the target gene was identified by sequencing the ends. Protein blast (blastp) assays indicated that it had high similarity to NAC67 from *Brachypodium*, rice and maize, and thus named *TaNAC67* (Accession No. KF646593). Sequence alignment, and similarities among species were determined by the MegAlign program in DNASTAR (DNASTAR, Inc). The secondary structure was predicted with PREDATOR (http://bioweb.pasteur.fr/seqanal/protein/intro-uk.html), and the functional region was identified using PROSITE (http://expasy.hcuge.ch/sprot/prosite.html). Subcellular localization was predicted with ProtComp v9.0 software (http://linux1.softberry.com/berry.phtml?topic=protcomppl&roup=programs&subgroup=proloc).

### Genetic characterization of *TaNAC67*

To identify the genomic origin and analyze the structure of *TaNAC67*, a pair of primers, flaking the open reading frame (ORF), was designed (GF, 5′- CGGAGAAGCAGGAAGCGGCAAT -3′, GR, 5′- ATAAATCCTGAACAAATGTGAACTATC -3′). The genomic fragments of *TaNAC67* were amplified using TransStart FastPfu Taq DNA polymerase and ligated into a pEASY-Blunt cloning vector (TransGen Biotech, CN), and then sequenced with a DNA analyzer (ABI 3730XL). The sequences were then analyzed with the MegAlign program in DNAStar software.

To isolate the promoter of *TaNAC67*, the genomic DNA sequence of *TaNAC67* was subjected to blastn to a genomic sequence database for *Ae. tauschii* (DD), the diploid D-genome donor of common wheat [50]. The hit scaffold with highest similarity to the query sequence was selected, and gene-specific primers (covering the upstream promoter and partial coding regions) were designed. The promoter regions were isolated by PCR, and the *cis*-acting regulatory elements were predicted by Plantcare (http://bioinformatics.psb.ugent.be/webtoolss/plantcare.htlm) [51].

### Phylogenetic tree construction of TaNAC67

Phylogenetic analysis was performed to understand the relationship between TaNAC67 and NAC members from wheat and other plant species. A maximum likelihood tree was constructed using putative protein sequences in the promlk program in the PHYLIP (version 3.69) software package with the bootstrap parameter set as 100.

### Quantitative real-time PCR

The cDNA templates for quantitative real-time PCR (qRT-PCR) were obtained as previously described [3]. The qRT-PCR was performed in triplicate with an ABI PRISM® 7900 system using the SYBR Green PCR master mix kit (Applied Biosystems Inc., USA) according to the manufacturer's instructions. *Tubulin* transcript was used to quantify the relative transcript level. Oligonucleotides of qRT-PCR primers were: QF, 5′-TGGTGGCGGACTACCTCTG-3′; QR, 5′-CCCGCGGCGTGAAGAAGT-3′. Relative gene expression was estimated using the 2^−ΔΔCT^ method [52]. The *Actin* transcript of *Arabidopsis* was used to quantify the expression levels of *TaNAC67* in the transgenic lines. The oligonucleotides for abiotic stress responsive genes were listed in Table S2 in File S1 [53], [54].

### Subcellular localization of TaNAC67 protein

The full-length ORF of *TaNAC67* was fused upstream of the GFP gene and placed under control of the constitutive CaMV 35S promoter in the pJIT163-GFP expression vector to construct a 35S::*TaNAC67-GFP* fusion protein. Restriction sites were added to the 5′- and 3′ ends of the coding region by PCR, and the oligonucleotides for fusion GFP subcloning were: F, 5′-CTCT***AAGCTT***TCATCGGCAGCGGAGCGATT-3′ (*Hin*d III site in bold italics), R, 5′-CTCT***GGATCC***GCGGACACGGGGGGA-3′ (*Bam* HI site in bold italics). The PCR product was cloned into the pJIT163-GFP vector and transformed into live onion epidermal cells by biolistic bombardment. Subcellular location of TaNAC67 was detected by monitoring the transient expression of GFP in onion epidermal cells as described earlier [47].

### Generation of transgenic plants

The coding region of *TaNAC67* cDNA was amplified by RT-PCR with the primers for subcellular localization cloning and cloned into the pPZP211 vector as a GFP-fused fragment driven by the CaMV 35S promoter [55]. Transformation vectors harboring *35S*::*GFP* or *35S*::*TaNAC67*-*GFP* were introduced into *Agrobacterium* and transferred into wild type *Arabidopsis* plants by the dip transformation method [56]. Positive transgenic lines were firstly screened on kanamycin plates and then identified by fluorescence detection.

### Morphological characterization of transgenic plants

Transgenic plants (T3) were characterized for morphological changes under short-day photoperiods (12-h light/12-h dark) in a growth chamber with a constant temperature of 22°C. Root morphology was examined on MS medium solidified with 1.0% agar. T3 homozygous transgenic and WT seeds were germinated on MS medium and grown vertically for primary root length measurement (10 d) and counts of lateral root numbers (14 d). For biomass measurement, transgenic plants and two controls were planted in sieve-like plates filled with mixed soil (vermiculite: humus  = 1∶1) and cultured under well-watered conditions.

### Measurement of physiological indices

For physiological index measurement, six randomly selected T3 homozygous lines were used and seedlings at designated age were subjected to different index measurements. Chlorophyll content was measured with a chlorophyll meter at seedling stage (4-week-old) (SPAD 502 Plus, Konica Minolta Sensing, Inc., JP). Fully expanded rosette leaves were selected for chlorophyll content measurements. One measurement was made for each plant, and 20 plants were used for transgenic lines and controls. Measurements of chlorophyll contents under salt stress conditions were performed at designated times after applying 250 mM NaCl solution.

The water potential (WP) of *Arabidopsis* seedlings (5-week-old) grown under normal or water deficit conditions was measured with a water potential meter (WP4, Decagon Devices, Inc., USA). Measurements were taken in dew point mode at room temperature. *Arabidopsis* seedlings growing in the same container as described in the section of morphological assays were selected for WP assays. Five seedlings of each line were collected as a sample.

Osmostic potential (OP) and chlorophyll florescence were measured at seedling stage (4 weeks old). The OP was measured with a Micro-Osmometer (Fiske® Model 210, Advanced Instruments, Inc., USA), chlorophyll florescence was assayed with a portable chlorophyll fluorescence meter (OS 30P, Opti-Sciences Inc., USA), and cell membrane stability (CMS) was determined with a conductivity meter (DDS-1, YSI, USA) as described [3]. The maximum efficiency of photosystem II (PS II) photochemistry, *Fv/Fm*  =  (Fm−F0)/Fm, was deployed to assess changes of the photosynthetic potential under salt stress. The activity of PS IIwas measured when moderate drought stress occurred or after a twenty-hour-exposure to salinity stress (300 mM NaCl). The CMS under salt stress was measured after applying NaCl solution (300 mM) to soil-grown seedlings (4-week-old) for 20 h. For CMS under drought stress, 7-day-old seedlings were exposed to PEG-6000 solution (−0.5 MPa) for 12 h and then subjected to determination. Free proline was extracted and quantified from fresh tissues of well-watered seedlings (0.5 g) as earlier described [57].

Ion fluxes were measured noninvasively under salt-shock and salt-pretreatment conditions. For salt-shock treatment, net H^+^ and K^+^ fluxes were measured in the root apices of 5-d-old seedlings of WT and *TaNAC67* plants. The seedlings were preincubated in buffer (0.5 mM KCl, 0.1 mM MgCl_2_, 0.1 mM CaCl_2_, 0.2 mM Na_2_SO_4_, and 0.3 mM MES, pH 6.0) for 30 min and assayed in the same buffer containing 100 mM NaCl at pH 6.0. The transmembrane H^+^ and K^+^ fluxes in roots of *TaNAC67* transgenic plants (100 µm to root apex) were compared with WT. For salt-pretreatment experiments, 4-d-old seedlings were treated for 24 h on MS medium to which 100 mM NaCl had been added, and then the transmembrane Na^+^ fluxes were measured as described above. Ionic fluxes were calculated with mageflux, developed by the Xuyue Company (http://xuyue.net).

### Abiotic stress tolerance assays

Drought tolerance assay were performed on 5-week-old seedlings. After germination on MS plates, 7-d-old seedlings of transgenic lines were planted in sieve-like rectangular plates (3 cm deep) filled with a mixed soil (vermiculite: humus  = 1∶1) and well-watered. The seedlings were cultured in a greenhouse (22°C, 70% humidity, 120 µmol. m^−2^.s^−1^, 12 h light/12 h dark cycle) without watering. For salt tolerance assessments, *Arabidopsis* seedlings were cultured as for drought tolerance assays. Water was withheld for 3 weeks and plants were then well irrigated with NaCl solution (250 mM) applied at the bottom of the plates. When the soil was completely saturated with salt water, free NaCl solution was removed and the plants were cultured normally. For freezing tolerance evaluations, normally cultured *Arabidopsis* seedlings (4-week-old) were stressed in a −8±1°C freezer for 1.0 h, and then cultured at 15°C for 24 h to facilitate recovery, and finally cultured under normal growing conditions.

### Statistical Analysis

The data in the experiments of physiological index and abiotic tolerance assays were subjected to statistical analysis using the Duncan's Multiple Range Test program in Statistic Analysis System (SAS) (http://www.SAS.com).

## Results

### Sequence characterization of TaNAC67

The full-length cDNA of the target gene obtained by screening full-length wheat cDNA libraries was 1098 bp, including a 92 bp 5′ UTR, 894 bp open reading frame, and 112 bp 3′ UTR. The target gene was named *TaNAC67* based on the structural domains of the deduced protein and blastp results. The ORF encodes a polypeptide of 297 amino acid residues (AA) with a predicted molecular mass of 33.3 kD and pI of 5.41. The deduced amino acid sequence of TaNAC67 shows high homology with NAC family members of monocots, i.e. wheat, *Brachypodium distachyon*, rice, barley and maize, and lower homology with counterparts from dicot species, such as *Glycine max* and *A*. *thaliana*. TaNAC67 has identities of 90.2% to TaGRAB1 (CAA09371), 74.9% to BdNAC67 (XP_003557366), 70.7% to OsNAC67 (NP_001059213), 65.3% to ZmNAC67 (ACG31804), 61.2% to OsNAC1 (ABD52007), 60.5% to SbNAC1 (AFO85373), 56.1% to TaNAC2 (AAU08786), 54.1% to ZmNAC1 (ADK25055), and 39.1% to TaNAC69 (AAU08785).

**Figure 1:**
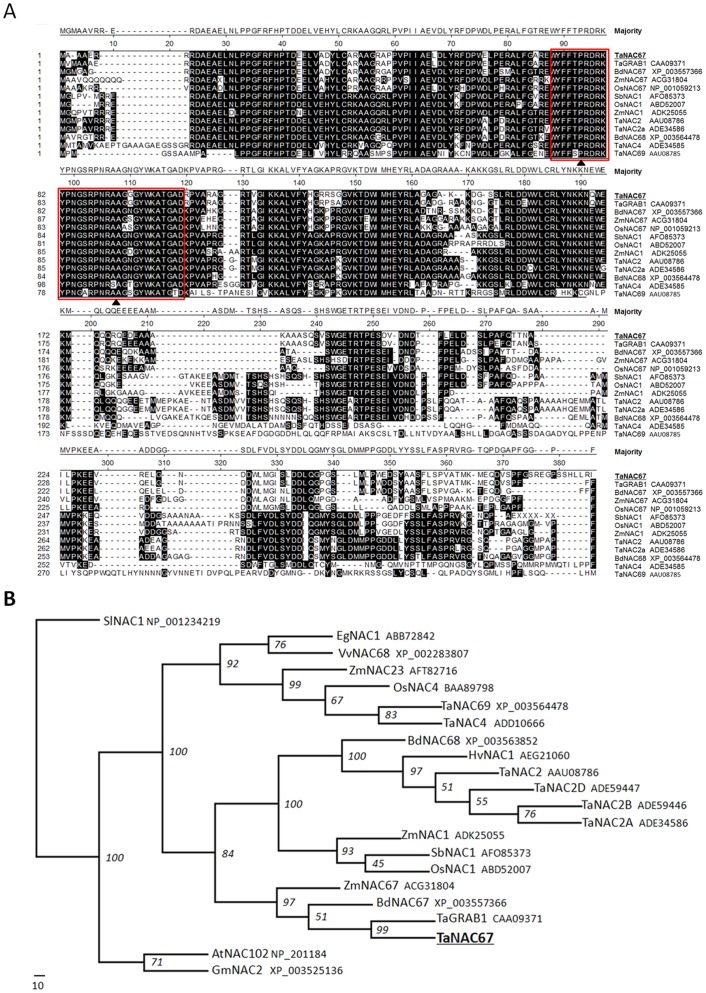
Sequence alignment of TaNAC67 and NACs in various plant species. A. Amino acid alignment of TaNAC67 and other NAC family members from selected plant species. The numbers on the left indicate amino acid position. Shared amino acid residues are in black background. Gaps, indicated by dashed lines are introduced for optimal alignment. The region underlined indicates the conserved NAC-domain. ▴, conserved amino acid motif (AA sequences in red rectangles). Alignments were performed using the Megalign program of DNAStar. B. Phylogenetic tree of TaNAC67 and NAC members from other plant species. Abbreviations: At, *Arabidopsis thaliana*; Bd, *Brachypodium distachyon*; Eg, *Elaeis guineensis*; Gm, *Glycine max*; Hv, *Hordeum vulgare*; Os, *Oryza sativa*; Sb, *Sorghum bicolor*; Sl, *Solanum lycopersicum*; Vv, *Vitis vinifera*; Zm, *Zea mays*. The phylogenetic tree was constructed with the PHYLIP 3.69 package, and the bootstrap values are in percent.

Scansite analysis indicated that TaNAC67 has two regions. The N-terminal region contains a NAC domain (18–168 AA) that is highly conserved across both monocots and dicots, and functions as a DNA binding domain (Fig. 1A). The C-terminal transcription activation region is remarkably divergent and involved in the regulation of downstream gene expression under various environmental stimuli [11], [58]. TaNAC67 fell into the stress-responsive NAC group based on the conserved NAC domain, and a highly conserved motif possibly involved in DNA binding was identified in the NAC domain (74-105AA) (Fig. 1A) [11]. The secondary structure prediction revealed that the TaNAC67 sequence formed 10 α-helixes and 3 β-pleated sheets.

A phylogenetic tree was constructed with the full-length amino acid sequences of TaNAC67 and some NAC family members from different species. TaNAC67 was clustered in a clade with TaGRAB1, BdNAC67, OsNAC67 and ZmNAC67 (Fig. 1B).

### Genetic characteristics of *TaNAC67*

**Figure 2:**
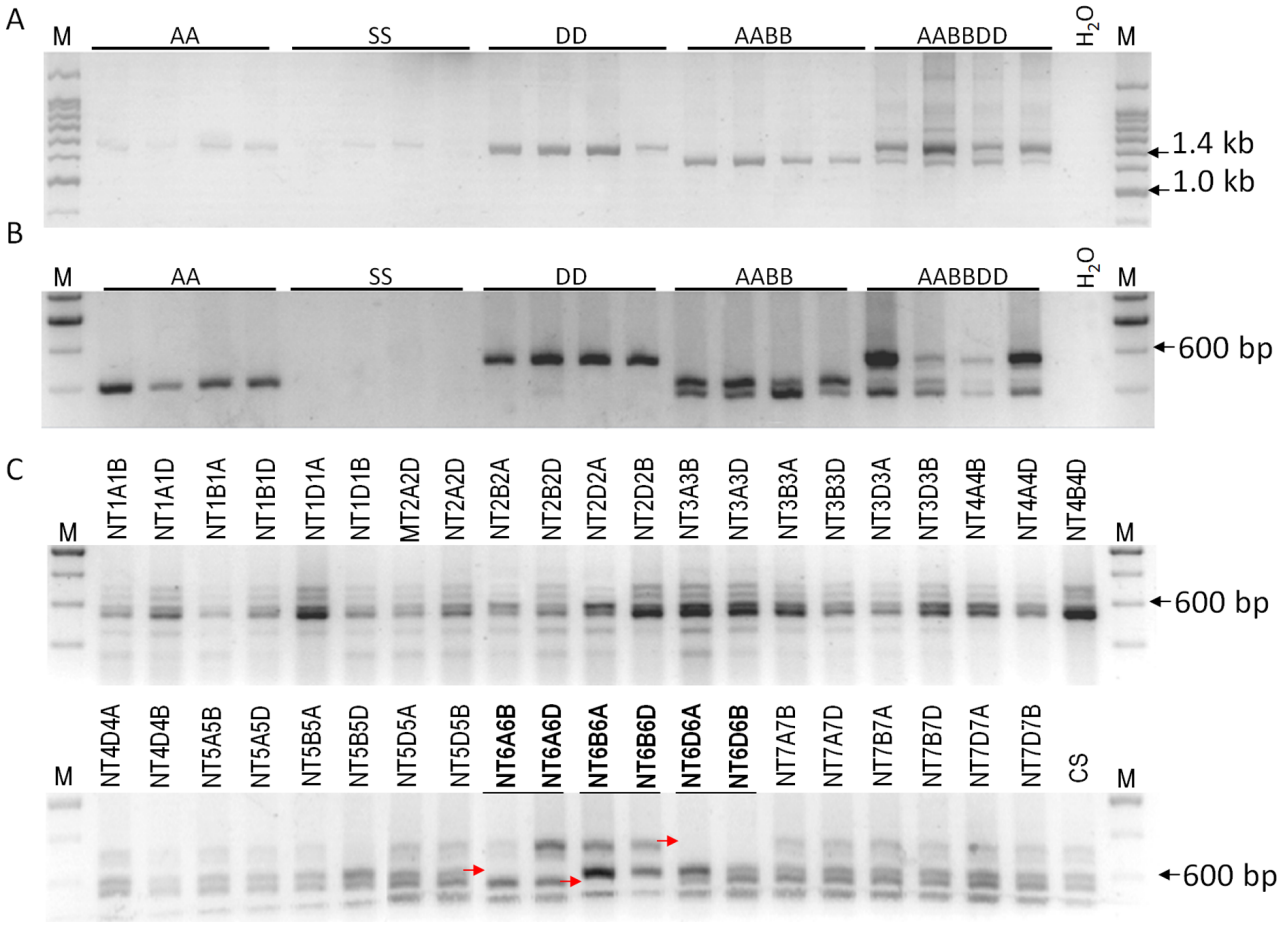
Chromosome location of *TaNAC67*. A. Genomic origin of *TaNAC67* among 20 accessions of wheat and related species. B. Three *TaNAC67* genes were identified in the A, B and D genomes of hexaploid wheat. AA, *T*. *urartu*; SS, *Ae*. *speltoides*; DD, *Ae*. *tauschii*; AABB, *T*. *diccocoides*; AABBDD, *T*. *aestvium*; M, 200 bp DNA ladder. C. Chromosome location of *TaNAC67* genes using 41 nulli-tetrasomic (NT) lines of Chinese Spring. Different *TaNAC67* genes were missing in each of the NT lines for homoeologous group 6. NT, nulli-tetrasomic line; M, 200 bp DNA ladder. Arrow, pointing at the missing bands in corresponding NT lines.

To identify the genomic origin of *TaNAC67*, 20 accessions of various wheat species were selected for PCR. DNA sequencing results showed that the target fragments were amplified in accessions carrying the A and D genomes. The two genes were 1.35 and 1.46 kb in size (Fig, 2A). The size difference between the two genes was attributed to a 110 bp insertion in the D genome member. Based on the sequence length polymorphisms, a primer locating in the upstream of the insertion was designed (IPF, 5′-GGTCTCCTGGGGTGAGACGC-3′). It amplified the polymorphic regions when combined with the GR primer (described in Materials and Methods) in wheat species with the A, D, AB or ABD genomes, but not in the putative S (B) genome donor species, suggesting that *Ae. speltiodes* differed from present day tetraploid and hexaploid wheat in regard to the locus in the B genome. One gene was amplified in A and D genomic diploid accessions, and two and three genes were present in the tetraploid and hexaploid wheat accessions, respectively (Fig. 2B). Using nulli-tetrasomic lines of Chinese Spring (CS), the three genes were located on chromosome 6A, 6B and 6D (Fig. 2C). Gene structure assays showed that *TaNAC67* contained one intron and two exons, and the insertion in the D genomic member was located in the intron. Sequence alignment showed the target cDNA sequences were identical with the coding region of the D genome member, demonstrating that *TaNAC67* was located on chromosome 6D.

A scaffold (No. 58115) with the highest identity to the query *TaNAC67* genomic sequence was obtained by blastn with D genomic sequence database [50]. Three pairs of gene-specific primers were designed according to the sequence, and one pair of primers (PF, 5′-GAATCATGGTCATTAATACTGCCATA-3′; PR, 5′-GTGGCGCGGGTAGAGAGAGAG-3′) that amplified the promoter sequence of the D genome allele was obtained. The fragment was about 2,200 bp in size. *Cis*-acting regulatory element analysis showed the presence of some basic components and stress-responsive element binding motifs in the promoter region (1,500 bp) included TATA boxes, CAAT boxes, abiotic stress response *cis*-elements (four ABA response elements, ABRE), and multiple biotic stress response elements (including two salicylic acid and three MeJA-responsive elements), one defense responsive element, and one gibberellin-responsive element (Table 1).

**Table 1:** *Cis*-acting regulatory elements identified in the promoter region of *TaNAC67* involved in response to biotic and abiotic stimuli.

Site name	Species	Position	Strand	Matrix score	Sequence	Function
**ABRE**	*O. sativa*	590	-	9	GCCGCGTGGC	*cis*-acting element involved in the abscisic acid responsiveness
	*Hordeum vulgare*	1432	+	9	CGTACGTGCA	*cis*-acting element involved in the abscisic acid responsiveness
	*A. thaliana*	1157	+	6	CACGTG	*cis*-acting element involved in the abscisic acid responsiveness
	*A. thaliana*	1434	+	6	CACGTG	*cis*-acting element involved in the abscisic acid responsiveness
**TC-rich repeats**	*Nicotiana tabacum*	846	+	9	ATTTTCTTCA	*cis*-acting element involved in defense and stress responsiveness
**GARE-motif**	*Brassica oleracea*	635	-	7	AAACAGA	gibberellin-responsive element
**TCA-element**	*B. oleracea*	738	-	9	CAGAAAAGGA	*cis*-acting element involved in salicylic acid responsiveness
	*B. oleracea*	1479	+	9	GAGAAGAATA	*cis*-acting element involved in salicylic acid responsiveness
**TGACG-motif**	*H. vulgare*	429	+	5	TGACG	*cis*-acting regulatory element involved in MeJA-responsiveness
	*H. vulgare*	1008	-	5	TGACG	*cis*-acting regulatory element involved in MeJA-responsiveness
	*H. vulgare*	1005	+	5	TGACG	*cis*-acting regulatory element involved in MeJA-responsiveness
	*H. vulgare*	1031	+	5	TGACG	*cis*-acting regulatory element involved in MeJA-responsiveness

### Dynamic response of *TaNAC67* to abiotic stresses

**Figure 3:**
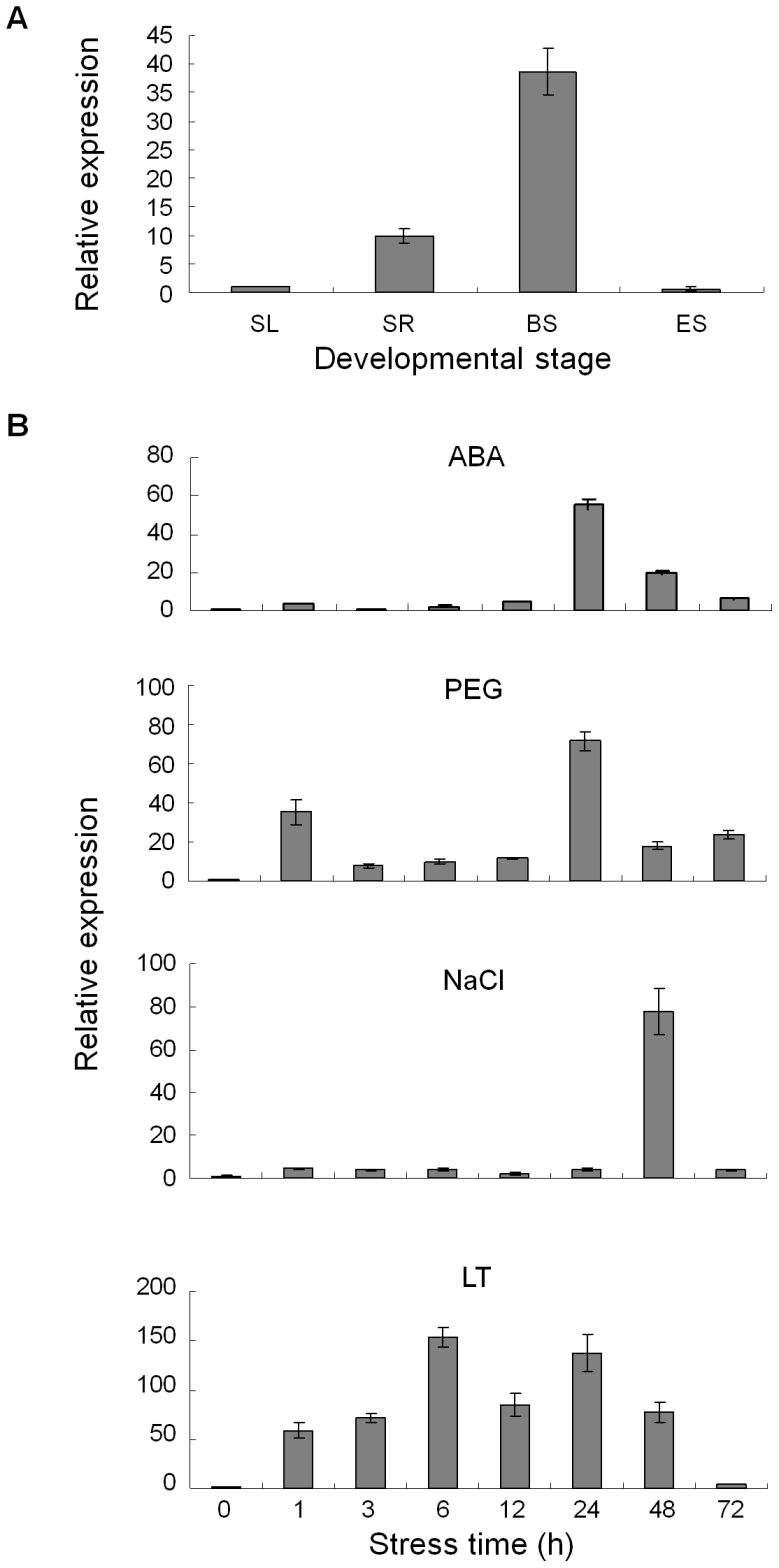
Expression patterns of *TaNAC67* in common wheat. Expression patterns of *TaNAC67* in different tissues at different developmental stages (A) and under stress treatments with ABA, PEG (−0.5 MPa), salt (NaCl) and low temperature (LT) (B). Two-leaf seedlings of common wheat cv. Hanxuan 10 were exposed to abiotic stresses as described in Materials and Methods. The 2^−ΔΔCT^ method was used to measure relative expression levels of the target gene in stressed and non-stressed leaves. Three samples were collected for each time point per treatment, and the experiments for each sample were triplicate. Means were generated from three biological replications; bars indicate standard errors. SL, seedling leaves; SR, seedling roots; BS, booting spindles; ES, emerging spikes; PEG, PEG-6000; LT, low temperature.

The expression of *TaNAC67* was identified by qRT-PCR in seedling leaves, seedling roots, booting spindles and emerging spikes; the highest expression occurred in booting spindles, with lower levels in seedling roots and leaves (Fig. 3A). Diverse expression levels of *TaNAC67* were recorded in seedling leaves of plants exposed to water deficit, salt, low temperature (LT) and ABA (Fig. 3B). *TaNAC67* was significantly activated by all four treatments. Among them, *TaNAC67* was sensitive to LT and PEG stresses at an early stage (1 h after exposure to abiotic stress), and sensitivity to ABA and NaCl occurred later. Both the expression patterns and maximum expression levels were fundamentally different for each type of stress. For example, the expression levels of *TaNAC67* peaked at 6 h for cold, 24 h for ABA and PEG, and 72 h for NaCl, and the corresponding maxima were 153, 55, 28 and 77-fold relative to the control.

### Subcelluar localization of TaNAC67

**Figure 4:**
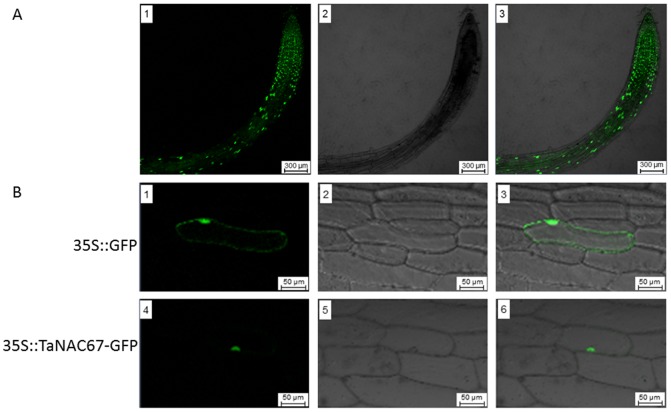
Subcellular localization of TaNAC67 in onion epidermal cells. A. The construct harboring 35S::TaNAC67-GFP was introduced into *Agrobacterium*, and transferred into *Arabidopsis* by floral infiltration. Positive transgenic lines were screened with kanamycin, and then examined with a confocal microscope. Images are dark field for green fluorescence (1), and root outlook (2) and combined in bright field (3). Scale bar  = 300 µm. B. Cells were bombarded with constructs carrying GFP or TaNAC67-GFP as described in Materials and Methods. GFP and TaNAC67-GFP fusion proteins were transiently expressed under control of the CaMV 35S promoter in onion epidermal cells and observed with a laser scanning confocal microscope. Images were taken in dark field for green fluorescence (1, 4), cell outlook (2, 5) and combination (3, 6) in bright field. Scale bar  = 100 µm. At least 30 cells containing each construct were examined.

Prediction of subcellular localization using ProtComp v6.0 software suggested that TaNAC67 was a typical cell nucleus localization protein. To identify the cellular localization of TaNAC67, we examined the expression and distribution of TaNAC67 in both transgenic *Arabidopsis* root and onion epidermal cells by expression of the fusion protein with GFP by fluorescence microscopy. As Fig. 4A showed, TaNAC67-GFP was expressed in transgenic *Arabidopsis* roots, especially in young root tips. To further identify the exact cellular localization, we determined the distribution of GFP-tagged TaNAC67 in onion epidermal cells by means of fluorescence microscopy (Fig. 4B). TaNAC67 was observed only in the cell nucleus, demonstrating that it interacted with the cell nucleus.

### Morphological characteristics of *TaNAC67*-overexpressing *Arabidopsis* plants

Six transgenic lines were randomly selected, and significantly different expression levels of *TaNAC67* were identified, the highest expression was observed in L4, followed by L2, L6, L5 L3 and L1 (Fig. S1 in File S1). To evaluate the applicability of *TaNAC67* in transgenic breeding for abiotic stress tolerance, the phenotypes of *TaNAC67* plants at different developmental stages were characterized. The primary roots of five of the six *TaNAC67* lines were significantly shorter than the WT and GFP controls for 10-day old seedlings (Fig. S2A, 2B in File S1), and there was a negative relationship between expression level and primary root length. However, no differences in germination rates (data not shown) and lateral root numbers were identified (Fig. S2C in File S1). For seedlings grown both on MS medium and in soil, there were no differences in seedling size or in fresh and dry weights between the transgenics and WT plants (Fig. S3 in File S1).

### *TaNAC67* transgenics have improved physiological traits for abiotic stress tolerance

**Figure 5:**
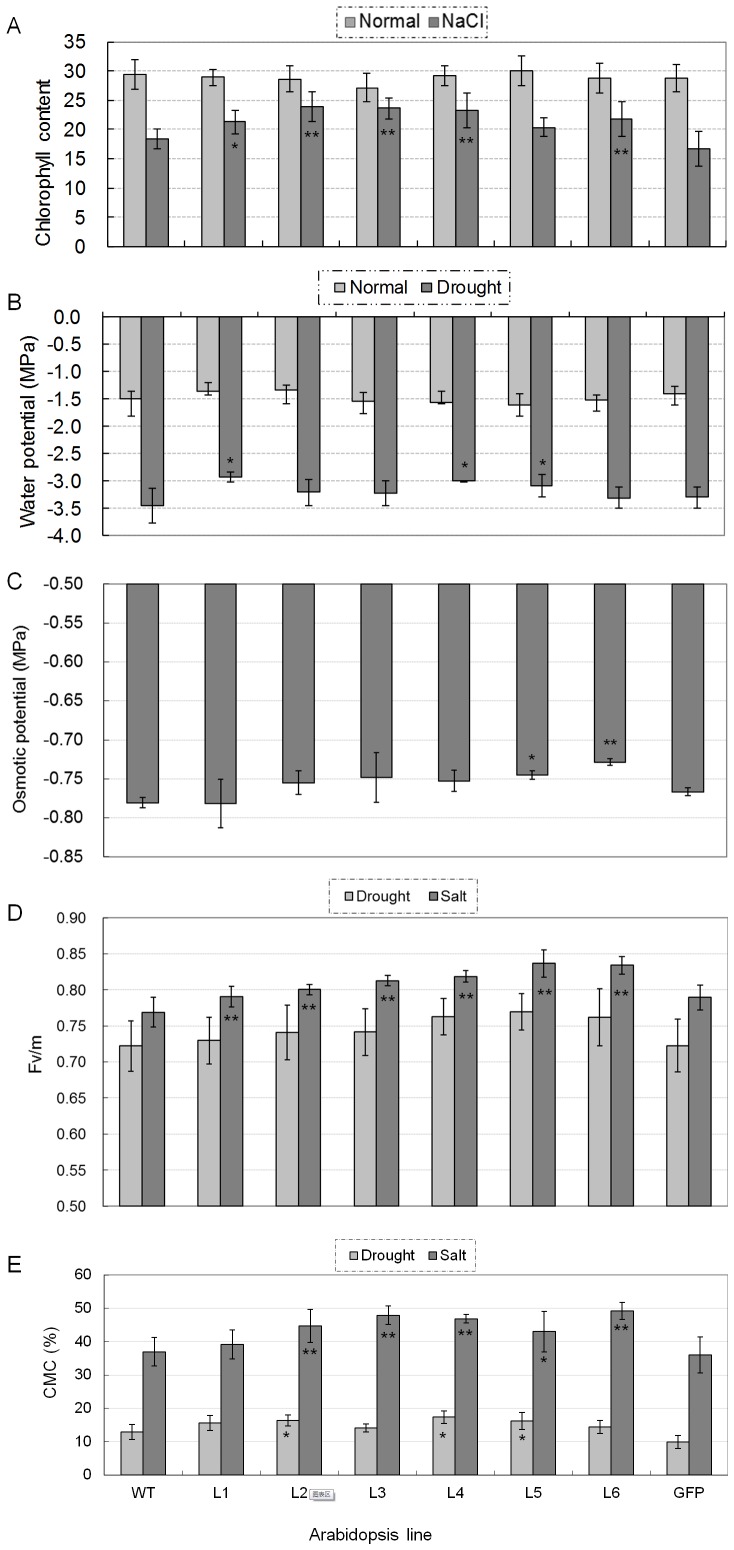
Comparisons of physiological indices related to abiotic stress response of *TaNAC67* transgenics under normal or/and stress conditions. A. Most *TaNAC67* transgenics had higher chlorophyll contents relative to control plants under salt stress. Values are means ± SE (n = 20 plants). *,** significantly different from WT at *P* = 0.05, 0.01, respectively. B. *TaNAC67* transgenics had higher water potentials than the controls. Six *TaNAC67* transgenic lines and controls grown in the same containers were subjected to water potential assays performed under well-watered and drought stress conditions. Five plants of each line were collected as one sample, and the experiment consisted of four replications. Values are means ± SE. C. Transgenic *TaNAC67* plants had higher osmotic potentials than the controls. Six *TaNAC67* transgenic lines, and WT and GFP controls, cultured under well-watered conditions, were subjected to osmotic potential assays. Five plants of each line were pooled as a sample, and the experiment consisted of three replications. Values are means ± SE. D. Comparison of photosynthetic potentials of *TaNAC67* transgenics and controls after exposure to high salinity and drought stress. The *Fv/Fm* ratios of five transgenics were significantly higher than the two controls. The youngest fully expanded leaves were selected to determine chlorophyll fluorescence; three measurements were made for each plant, and 20 plants were used for WT and transgenic lines. Values are means ± SE (n = 20 plants). E. *TaNAC67* transgenics have enhanced CMS relative to control plants after exposure to salt stress and water deficit (PEG-6000, −0.5 MPa). Fifteen seedlings were pooled as a sample; three samples were measured for each line. Values are means ± SE.

Chlorophyll content is an important parameter for salt tolerance assessment in plants. There was no difference between *TaNAC67* transgenics and WT *Arabidopsis* plants in chlorophyll content under normal growing conditions. The chlorophyll contents changed dynamically with a rapid decrease, slow increase and slow decrease pattern after exposure to high salinity (Fig. S4 in File S1). The final chlorophyll contents for five of the six transgenic lines were significantly higher than the WT and GFP controls at 72 h (Fig. 5A).

To assess water retention abilities of transgenic *Arabidopsis*, the transgenic lines, along with WT and GFP controls were grown under normal and moderate drought stress conditions. There were no differences in WP between the controls and transgenics under normal growing conditions, whereas the WP of the transgenics were higher than the controls under moderate drought stress (when the rosette leaves became dark); differences for three of the six transgenics were significant, indicating that the transgenic lines retained more water than the controls (Fig. 5B).

To reveal the physiological effects of *TaNAC67* overexpression, the six transgenic lines grown under well-watered conditions were selected for osmotic potential assays. The results indicated that the OPs of the transgenic lines were higher than those of WT and GFP under normal growing conditions, and the differences for two lines were significant (Fig. 5C). There were no differences in free proline content between transgenics and controls (Fig. S5 in File S1).

To evaluate photosynthesis potential of transgenics, chlorophyll fluorescence was measured. There were no differences between *TaNAC67* transgenics and WT in *Fv*/*Fm* (*Fv/m*) ratios under normal growing conditions (data not shown), whereas the *Fv/m* ratios of all *TaNAC67* lines under salt and drought stress conditions were higher than the two controls. The differences between transgenics and controls were not significant under drought stress, however, the differences for five of the six lines reached significant levels under salt stress (Fig. 5D).

To determine the response of *TaNAC67* plants under high salinity and water deficit, the transgenics were exposed to drought and salt stresses for cell membrane stability assessment. The CMS of all the transgenic lines were higher than the controls under the two stress conditions, and five of the six comparisons were significant for salt stress, whereas three of six comparisons were significant for drought (Fig. 5E).

**Figure 6:**
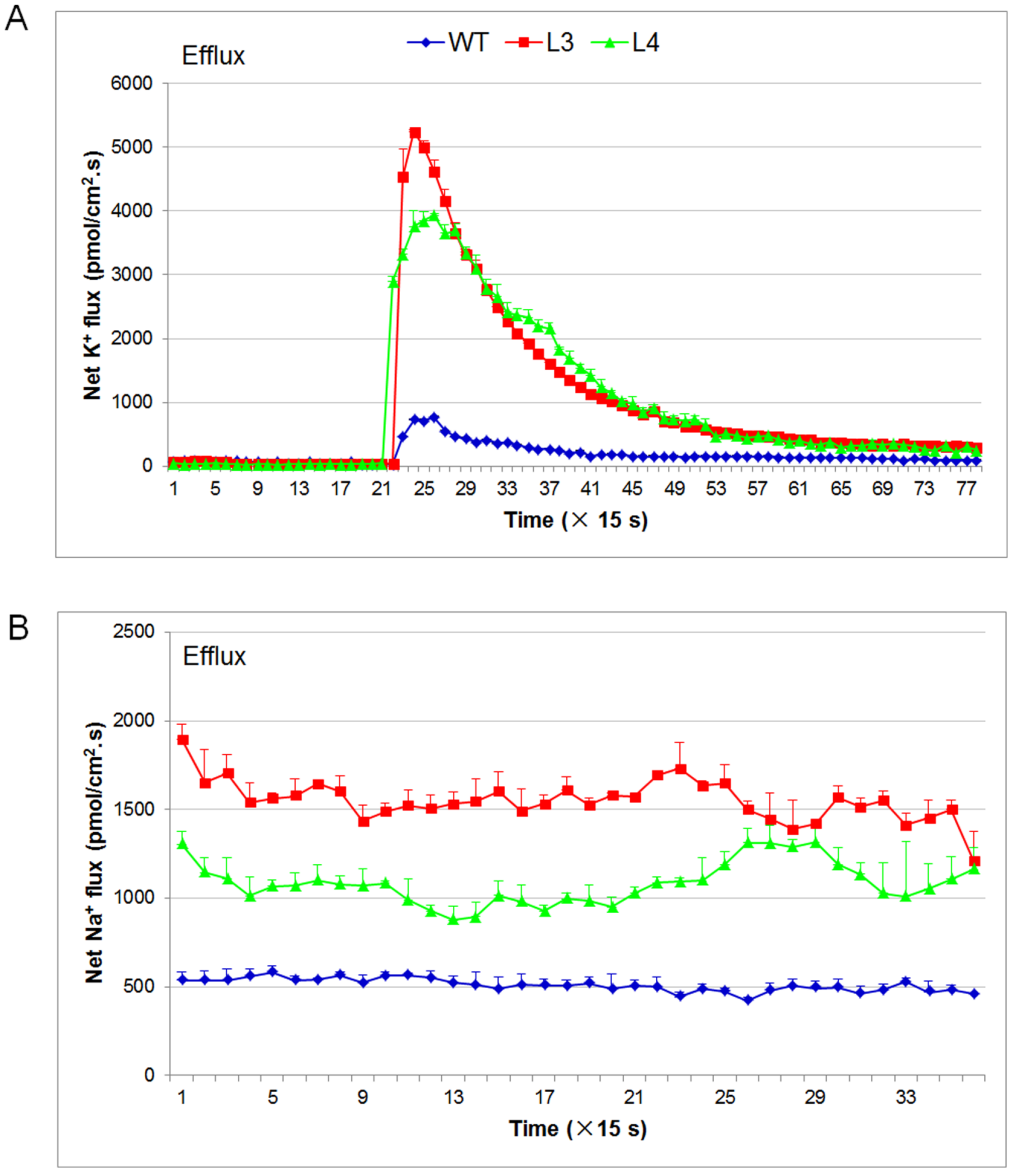
*TaNAC67* transgenics have higher K^+^ and Na^+^ ion efflux rates than WT. A. The transgenics had a higher K^+^ ion efflux rate than WT plants after a 30 min NaCl shock. *Arabidopsis* seedlings were pre-incubated in buffer (0.5 mM KCl, 0.1 mM MgCl_2_, 0.1 mM CaCl_2_, 0.2 mM Na_2_SO_4_, and 0.3 mM MES, pH 6.0) for 30 min and assayed in the same buffer containing 100 mM NaCl at pH 6.0. Five plants were measured for each line. Values are means ± SE. B. *TaNAC67* transgenics had higher Na^+^ ion efflux rates after treatment with 100 mM NaCl. *Arabidopsis* seedlings were pretreated on MS medium with 100 mM NaCl for 24 h, and then subjected to measurement of Na^+^ ion flux rates. Five plants were measured for each line, and the values are means ± SE.

To decipher the mechanism of salt tolerance in *TaNAC67* transgenics, H^+^ and K^+^ flux rates were measured after a 100 mM NaCl shock. Significantly higher K^+^ ion efflux rates were identified in the transgenics than in WT (Fig 6A). However, there was no difference in H^+^ flux rate (data not shown). To further disentangle the underline mechanism, Na^+^ ion flux rates were measured in plants pretreated with salt. The Na^+^ ion efflux rates were significantly higher in transgenic lines than in WT control (Fig 6B).

### *TaNAC67* transgenics have pronounced tolerances to multiple abiotic stresses

**Figure 7:**
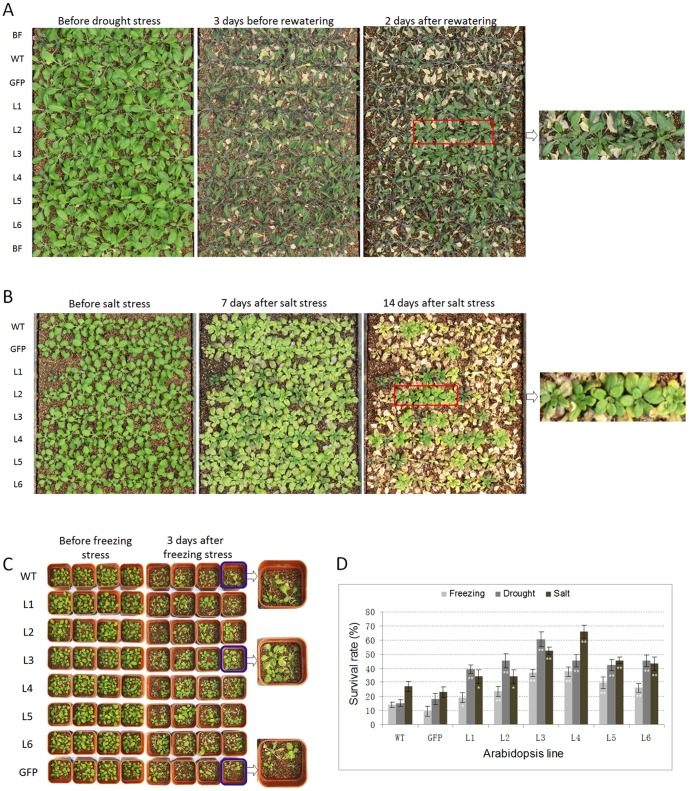
Transgenic *TaNAC67 Arabidopsis* has enhanced tolerance to drought, salt and freezing stresses. Phenotypes of six *TaNAC67* transgenics and WT and GFP controls following drought stress (A), salt stress (B), and freezing stress (C). D, survival rates of transgenic lines under different abiotic stresses. For drought and salinity stress, 11 plants were grown for each line in one treatment, and triplication occurred in three separate plates. For freezing stress, normally pot-cultured transgenic seedlings at 4 weeks were divided into three parts, and each part was stressed at −8±1°C for 1.5 h; 20 plants (5 pots) of each line were used for an experiment. L1-6, six transgenic lines; BF, buffer line. *, ** significantly different at *P* = 0.05, 0.01, respectively. Values are means ± SE.

To characterize the performance of *TaNAC67* plants under drought stress in soil, transgenic lines were subjected to drought tolerance assays. After a 30-day-water-withholding period, the rosette leaf colors of WT and GFP plants became dark, whereas the transgenics were still green. On the 35th day, all the WT and GFP plants displayed signs of severe wilting (with all rosette leaves severely curled and some leaf death) compared to less wilting for the *TaNAC67* transgenics (Fig. 7A). After rewatering for two days, 40–60% of transgenic plants survived, significantly higher than control plants (12–18%) (Fig. 7A, D).

The leaf tips of WT *Arabidopsis* began to crimple 24 h later after exposing to salinity stress, but there was no evident crimpling on the transgenics. Seven days later, clear differences were evident (Fig. 7B). The transgenics were much greener than the controls. Two weeks later, only 28% of WT and 22% of GFP plants were alive; the survival rates of transgenics were 35–65%, significantly higher than the controls, suggesting *TaNAC67* plants had enhanced tolerance to salt stress (Fig. 7B, D).

After exposing to severe freezing stress, 18–38% of transgenic plants survived, whereas the survival rates of the two controls were only 8–13%, significantly lower than transgenics (Fig. 7C, D).

### Enhanced expression of abitotic stress responsive genes in *TaNAC67* plants

**Figure 8:**
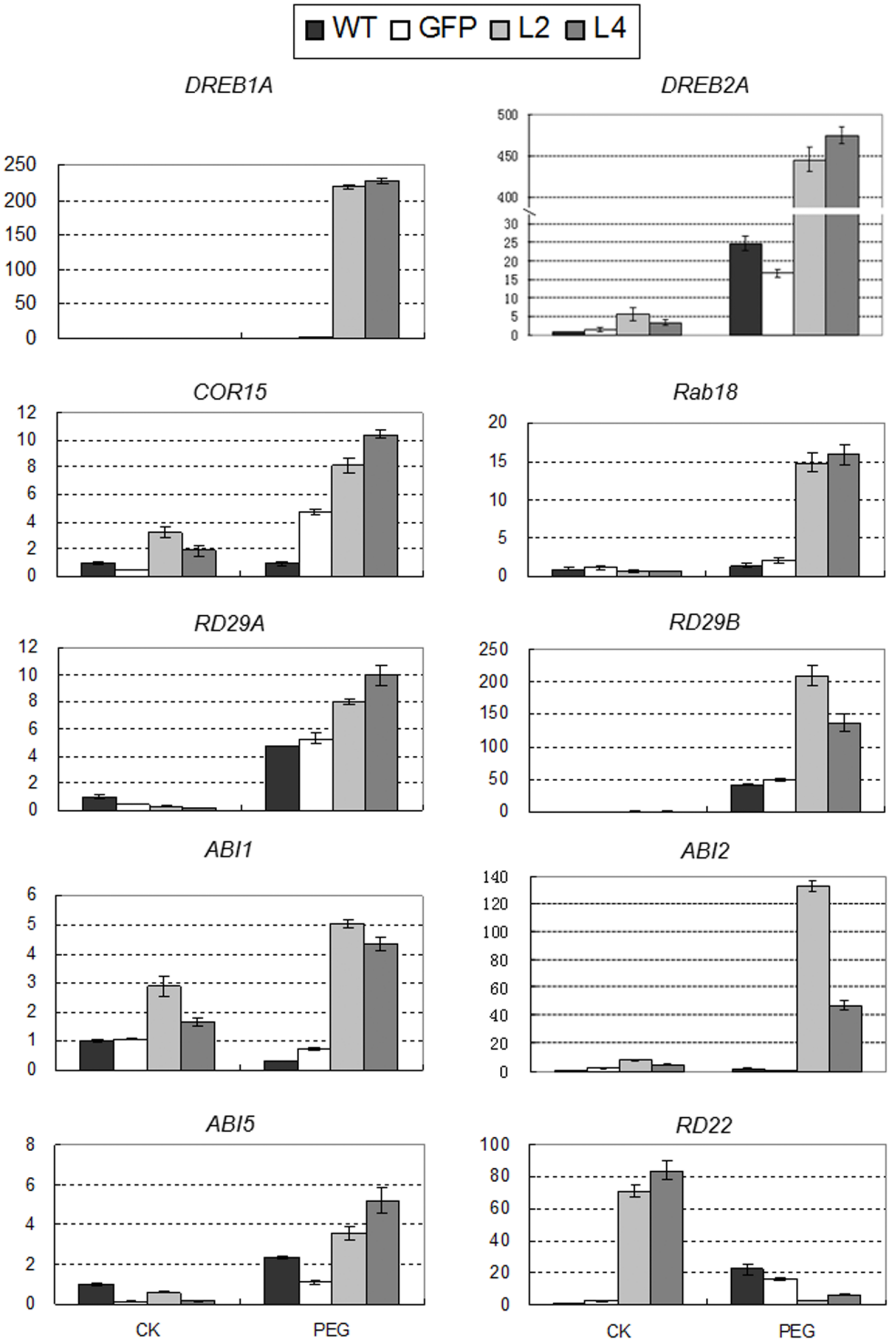
Comparisons of relative transcript levels of *DREB1A*, *DERB2A*, *RD29A*, *RD29B*, *Rab18*, *Cor15*, *RD22 ABI1*, *ABI2* and *ABI5* in WT and vector control and *TaNAC67* overexpressing lines treated for 3 h with PEG-6000 (−0.5 MPa) and assessed by qRT-PCR. Seedlings harvested before water deficit stress were used as control (CK). Ten seedlings were pooled as a sample, three samples were prepared for qRT-PCR on each line, and the experiments were triplicate. Vertical columns indicate relative transcript levels. Values (and error bars) were calculated using data from three independent assays.

Morphological assays indicated that the *TaNAC67* transgenics had enhanced tolerance to drought, salt and cold stresses. To probe the underlying molecular mechanisms, ten abiotic stress responsive genes, viz. *DREB1A*, *DREB2A*, *CBF1*, *CBF2*, *RD29A*, *RD29B*, *RD22*, *COR15*, *COR47* and *Rab18*, and four ABA synthesis or response genes, viz. *ABA1*, *ABI1*, *ABI2* and *ABI5*, were selected to determinate their expression levels in two transgenic lines, L3 and L4 (with second lowest and highest expression levels of *TaNAC67*, respectively) under water deficit or non- stressed conditions. The qRT-PCR results showed that transcripts of four genes (*DREB2A*, *COR15*, *ABI1* and *ABI2*) were significantly and consistently higher in both stressed and non-stressed transgenic plants, and expression levels of five genes (*DERB1A*, *RD29B*, *RD29A*, *Rab18* and *ABI5*) were significantly higher in PEG-stressed *Arabidopsis* plants (Fig. 8); whereas the transcript levels of the other four genes (*CBF1*, *CBF2*, *COR47* and *ABA1*) did not differ significantly (data not shown). Interestingly, the expression levels of *RD22* increased in non-stressed transgenic plants, and were much lower in PEG-stressed transgenic plants.

## Discussion

### TaNAC67 is a novel member of NAC family

Sequence analysis showed TaNAC67 has high identity with reported stress responsive NAC members in the N-terminal DNA binding domains, including OsNAC1, TaGRAB1, TaNAC2, TaNAC2a, TaNAC4 and TaNAC69, while differs in the C-terminal transcriptional activation domain. The difference might contribute to functional diversity in plants. Although the DNA binding domains of TaNAC67 and TaNAC2 are quite similar, the characteristics of the two encoding genes differ remarkably. Firstly, their expression levels and response times are different under abiotic stress conditions [3]. Secondly, overexpression of *TaNAC67* leads to shorter primary roots (Fig. S2A in File S1), while *TaNAC2* transgenics have longer primary roots relative to WT [3], and a negative relationship is evident between primary root length and the expression level of *TaNAC67* in *Arabidopsis* (Fig. S1, S2A in File S1), thus favorable expression level of *TaNAC67* should be reconsidered following selection of a proper promoter for abiotic stress tolerance enhancement. Thirdly, the abiotic stress responsive genes regulated by the two genes are different. Overexpression of the two genes leads to the upregulation of *DREB2A*, *RD29A*, *RD29B*, *Rab18*, *ABI1, ABI2* and *ABI5* in *Arabidopsis*, while *TaNAC67* also upregulates the expression of *DREB1A* and *COR15*. Moreover, the expression pattern of dehydration responsive gene *RD2*2 is different in the two transgenics. *RD22* was upregulated under normal growth conditions in *TaNAC67* transgenics, and downregulated after exposure to drought stress. However, *RD22* was consistently upregulated in *TaNAC2* plants under both normal and stressed conditions [3]. Therefore, we speculate that the molecular mechanisms for abiotic stress tolerances enhancement for the two NACs might be different in *Arabidopsis*.

No differences in lateral root numbers were identified between transgenic plants and the controls, and no growth retardation was apparent in *TaNAC67* transformants (Fig. S2B, S3 in File S1), suggesting that *TaNAC67* has potential to improve tolerances to abiotic stresses in crops.

### Physiological changes in transgenic *TaNAC67* plants under various conditions

Environmental stresses often cause physiological changes in plants. Physiological indices such as CMS, OP, WP, chlorophyll content, chlorophyll fluorescence, Na^+^ and K^+^ flux rates are useful parameters for evaluating abiotic stress resistance in crop plants.

Chloroplasts are among the most sensitive organs responding to salt stress. Chlorophyll degeneration in crops often occurs rapidly after exposure to high salinity, and thus chlorophyll content could be an indicator of damage to the photosynthetic system by salt stress. Chlorophyll fluorescence of intact leaves, especially the fluorescence induction pattern, is a reliable, non-invasive method for monitoring photosynthetic events and reflects the physiological status of plants [59]. The ratio of variable to maximal fluorescence is an important parameter used to assess the physiological status of PSII. Environmental stresses that affect PS II efficiency are known to cause decreases in *Fv/m* ratio [60]. In the present research, we measured the two indices, and most transgenic lines were superior to the controls (Fig. 5A, D), suggesting that *TaNAC67* transgenics had more robust photosynthetic capabilities than the controls under severe salt stress conditions (Fig. 7B, D).

WP is a direct reflection of water retention ability for plants under water deficit conditions, and a higher WP means that more water is retained in plants. The WP of most *TaNAC67* transgenics were significantly higher than the WT and GFP controls under moderate drought stress conditions (Fig. 5B; Fig. 7A), suggesting that the transgenics have stronger water retention capacity. Osmotic potential is another direct indicator of water content in plants. Generally, a higher OP suggests more water retained in plant cell under normal or slight water stress conditions. The higher OPs of transgenics suggest that *TaNAC67* plants hold more water in plants, which is consistent with the WP results (Fig. 5C). Under our growing conditions, both the depth of container and soil moisture are fixed, thus the higher WP in transgenics had no relationship with root system but enhanced water retention ability, despite that the primary root length for most transgenic lines was significantly shorter than WT (Fig. S2B in File S1), therefore, we speculate the transgenics might have lower stomatal conductance under slight drought stress. In this regard, TaNAC67 might have a similar function with the well-characterized SNAC1 [37].

Cell membranes are among the first targets of many stresses. The maintenance of membrane integrity and stability under water stress conditions is a major component of environmental stress tolerance in plants [61], and CMS has been used for assessing tolerance to frost, heat and desiccation [62]. In this study, the CMS of *TaNAC67* plants under salinity and drought stresses were higher than the WT and GFP controls (Fig. 5E), demonstrating that CMS enhancement is caused by overexpression of *TaNAC67*, indicating that *TaNAC67* transgenics might have a strong capacity to tolerate adverse environmental stresses, as verified by the phenotyping assays in *Arabidopsis* (Fig. 7).

The ion flux measuring technique provides a unique possibility to link genetic/genomic data and cellular physiological behavior because of its non-invasive, high spatial and temporal resolution features [63]. NaCl-induced K^+^ efflux was demonstrated as a physiological ‘marker’ for salt tolerance in several species, including maize [64], barley [65], wheat [66] and *Arabidopsis* [63]. The ability to retain K^+^ most effectively (i.e. to minimize K^+^ efflux when Na^+^ was applied) proved to be a trait strongly correlated with ability to thrive at high salt concentrations in barley, and K^+^ flux measurements were recommended as a screen for selecting salt tolerance in crop species [67]. In this study, we measured K^+^ efflux under salt-shock conditions, and found that the K^+^ efflux rates of *TaNAC67* transgenics were significantly higher than WT *Arabidopsis* (Fig. 6A), suggesting that the transgenics did not have a stronger capacity to retain K^+^ compared to WT [63], [66], [68], [69]. This seemed to be in conflict with the evident salt tolerant phenotypes for the transgenic lines (Fig. 7B, D). To further decipher the mechanism of enhanced salt tolerance in transgenics, Na^+^ ion efflux rates were measured under conditions of salt pre-treatment, and the Na^+^ efflux rates in the transgenics were significantly higher than the WT control (Fig. 6B), suggesting that *TaNAC67* plants had stronger capacity to extrude Na^+^ ions. Based on the phenotypes and results of ion flux measurements, we attribute the enhanced tolerance to high salinity mainly to robust Na^+^ exclusion in the transgenics. Therefore, for *TaNAC67* transgenic *Arabidopsis*, K^+^ retention ability seems to have a lower correlation with salt tolerance, and homeostasis between K^+^ and Na^+^ might be a more appropriate parameter to assess the phenotype under salt stress.

### Overexpression of *TaNAC67* enhanced expression of abiotic stress response genes in *Arabidopsis*

Considerable evidence indicates that plant NAC family members play critical roles in response to hyperosmotic stress. Evidence from *Arabidopsis* shows that AtNAC019, AtNAC055 and AtNAC072 confer enhanced tolerance to drought [26], and AtNAC2 is involved in response to various plant hormones and salt stress [27]. Overexpression of *SNAC1/OsNAC1*, *SNAC2/OsNAC6*, *OsNAC045* and *OsNAC063* enhances tolerance to multiple abiotic stresses [32]–[35], [37]. Overexpression of *GmNAC20* resulted in enhanced tolerances to salt and freezing, and *GmNAC11* overexpression leaded to salt resistance [70]. Recent studies showed that *TaNAC69-1*, *TtNAMB-2*, *TaNAC4* and *TaNAC8* were involved multiple abiotic or/and biotic stress responses in wheat [41]–[43], and overexpression of *TaNAC2*, *TaNAC2a* and *TaNAC69* led to a pronounced enhancement of tolerance to multi-abiotic stresses [3], [45], [46]. Here, we observed the dynamic expressions of *TaNAC67* under various abiotic stresses and found that overexpression of *TaNAC67* led to enhanced tolerance to drought, salinity and freezing stresses in *Arabidopsis*. In the present study, *TaNAC67* transgenics were exposed to severe drought, salt and freezing stresses. Both morphological and physiological evidence strongly demonstrated that transgenic lines had more pronounced tolerances to drought, salinity and freezing stresses than WT.

Based on the present data, we attribute the enhanced tolerance to abiotic stress conferred by *TaNAC67* to significantly and consistently increased expression of abiotic stress responsive genes, including *DREB2A*, *COR15*, *ABI1* and *ABI2*. *DREB2A* is a crucial regulatory element involved in drought response [71]. Its significant upregulation undoubtedly increased the expression levels of downstream drought stress responsive genes, and thus enhanced tolerance to drought and or other abiotic stresses, because of the widespread cross talk between different environmental stress response pathways [2], [72]. The *ABI1* and *ABI2* genes encode homologous type-2C protein phosphatases, involved in ABA signaling [73], and their high expression level might lead to upregulation of genes controlled by *ABI1* and *ABI2* in the ABA dependent pathway, and might enhance an integrated tolerance to multiple abiotic stresses. *DREB1A/CBF3* encodes a crucial TF, whose overexpression leads to enhanced tolerance to drought, salinity and cold stresses [74], [75]. *RD29A*, *RD29B* and *Rab18* are low molecular weight hydrophilic protein encoding genes [76], [77]; significant augmentation of hydrophilic proteins in tissue sap should be helpful for water retention under stressed conditions. The high expression levels of these important stress-responsive genes in transgenic plants under PEG-stressed conditions will benefit transgenic plant survival under severe abiotic stress conditions.

This study primarily concerned the morphological and physiological effects of *TaNAC67* overexpression in *Arabidopsis* under normal and adverse conditions. Comprehensive investigations to understand the functional enhancement of WP in wheat are ongoing, and the results are expected to increase our knowledge of the mechanism of water retention in plants.

## Supporting Information

File S1 Fig. S1, Expression levels of *TaNAC67* in different transgenic *Arabidopsis* lines. Gene expression levels of *TaNAC67* differed significantly in different transgenic *Arabidopsis* lines. L1 - 6, *TaNAC67* transgenic lines. The expression of *TaNAC67* in L1 was regarded as a standard for its lower expression level. Fig. S2, Comparison of primary root lengths and lateral root numbers for *TaNAC67* transgenics and the two controls. A. Phenotype of primary roots for *TaNAC67* transgenics. B. The primary root lengths of most transgenic plants were significantly shorter than the WT and GFP controls. C. Lateral root numbers of trangenics and controls were not significantly different. *Arabidopsis* plants were sown on MS plates solidified with 1.0% agar and cultured vertically in a greenhouse. Primary root length and lateral root numbers were measured after 10 d and 14 d, respectively. *, significantly different at *P* = 0.05. Values are means ± SE (n = 20). Fig. S3, No differences were identified in biomass of *TaNAC67* transgenics and two controls. *Arabidopsis* plants were grown in sieve-like rectangular containers filled with mixed soil (vermiculite: humus  = 1∶1) and cultured under well-watered conditions as described in Materials and Methods. Six plants were collected per sample for biomass measurement; four replications were set for each *Arabidopsis* line and the plants were harvested at five weeks. Single plant biomasses were calculated before and after treatment in an 80°C oven for 24 h. FW, fresh weight; DW, dry weight. Four replications were performed, and values are means ± SE. Fig. S4, A rapid decrease, slow increase and slow decrease pattern in chlorophyll content was identified Arabidopsis after exposure to high salinity. The chlorophyll contents were measured at designated times after exposure to 300 mM NaCl solution. Twenty plants were measured for each line. Values are means ± SE (n = 20). Fig. S5, No differences were identified in free proline contents for *TaNAC67* transgenics and the two controls. *Arabidopsis* plants were cultured as described in Materials and Methods. Five plants were collected as a single sample for measurement of free proline content. The experiment consisted of three replications. Values are means ± SE. *Table* S1, Plant materials used for identification of genomic origins. Table S2, Primer pairs used in quantitative real-time PCR in *Arabidopsis*.
(DOCX)

## References

[1] Qu L, Zhu Y (2006) Transcription factor families in *Arabidopsis*: major progress and outstanding issues for future research. Curr Opin Plant Biol 9: 544–549. 16877030 10.1016/j.pbi.2006.07.005

[2] Seki M, Narusaka M, Ishida J, Nanjo T, Fujita M, et al (2002) Monitoring the expression profiles of 7000 *Arabidopsis* genes under drought, cold and high-salinity stresses using a full-length cDNA microarray. plant J 31: 279–292. 12164808 10.1046/j.1365-313x.2002.01359.x

[3] Mao X, Zhang H, Qian X, Li A, Zhao G, et al (2012) TaNAC2, a NAC-type wheat transcription factor conferring enhanced multiple abiotic stress tolerances in *Arabidopsis* J Exp Bot. 63: 2933–2946. 10.1093/jxb/err462 PMC3350912 22330896

[4] Chen Y, Yang X, He K, Liu M, Li J, et al (2006) The MYB transcription factor superfamily of *Arabidopsis*: expression analysis and phylogenetic comparison with the rice MYB family. Plant Mol Biol 60: 107–124. 16463103 10.1007/s11103-005-2910-y

[5] Mao X, Jia D, Li A, Zhang H, Tian S, et al (2011) Transgenic expression of *TaMYB2A* confers enhanced tolerance to multiple abiotic stresses in *Arabidopsis.* . Funct Integr Genomics 11: 445–465. 21472467 10.1007/s10142-011-0218-3

[6] Mukhopadhyay A, Vij S, Tyagi AK (2004) Overexpression of a zinc-finger protein gene from rice confers tolerance to cold, dehydration, and salt stress in transgenic tobacco. Proc Natl Acad Sci U S A 101: 6309–6314. 15079051 10.1073/pnas.0401572101 PMC395965

[7] Shen H, Liu C, Zhang Y, Meng X, Zhou X, et al (2012) OsWRKY30 is activated by MAP kinases to confer drought tolerance in rice. Plant Mol Biol 80: 241–253. 22875749 10.1007/s11103-012-9941-y

[8] Shinozaki K, Yamaguchi-Shinozaki K, Seki M (2003) Regulatory network of gene expression in the drought and cold stress responses. Curr Opin Plant Biol 6: 410–417. 12972040 10.1016/s1369-5266(03)00092-x

[9] Zhu J (2002) Salt and drought stress signal transduction in plants. Annu Rev Plant Biol 53: 247–273. 12221975 10.1146/annurev.arplant.53.091401.143329 PMC3128348

[10] Ooka H, Satoh K, Doi K, Nagata T, Otomo Y, et al (2003) Comprehensive analysis of NAC family genes in *Oryza sativa* and *Arabidopsis thaliana.* . DNA Res 10: 239–247. 15029955 10.1093/dnares/10.6.239

[11] Fang Y, You J, Xie K, Xie W, Xiong L (2008) Systematic sequence analysis and identification of tissue-specific or stress-responsive genes of NAC transcription factor family in rice. Mol Genet Genomics 280: 547–563. 18813954 10.1007/s00438-008-0386-6

[12] Le DT, Nishiyama R, Watanabe Y, Mochida K, Yamaguchi-Shinozaki K, et al (2011) Genome-wide survey and expression analysis of the plant-specific NAC transcription factor family in soybean during development and dehydration stress. DNA Res 18: 263–276. 21685489 10.1093/dnares/dsr015 PMC3158466

[13] Hu R, Qi G, Kong Y, Kong D, Gao Q, et al (2010) Comprehensive analysis of NAC domain transcription factor gene family in *Populus trichocarpa.* . BMC Plant Biol 10: 145. 20630103 10.1186/1471-2229-10-145 PMC3017804

[14] Souer E, van Houwelingen A, Kloos D, Mol J, Koes R (1996) The no apical meristem gene of *Petunia* is required for pattern formation in embryos and flowers and is expressed at meristem and primordia boundaries. Cell 85: 159–170. 8612269 10.1016/s0092-8674(00)81093-4

[15] Zhong R, Demura T, Ye Z (2006) SND1, a NAC domain transcription factor, is a key regulator of secondary wall synthesis in fibers of *Arabidopsis.* . Plant Cell 18: 3158–3170. 17114348 10.1105/tpc.106.047399 PMC1693950

[16] Zhong R, Richardson EA, Ye Z (2007) Two NAC domain transcription factors, SND1 and NST1, function redundantly in regulation of secondary wall synthesis in fibers of *Arabidopsis.* . Planta 225: 1603–1611. 17333250 10.1007/s00425-007-0498-y

[17] Mitsuda N, Ohme-Takagi M (2008) NAC transcription factors NST1 and NST3 regulate pod shattering in a partially redundant manner by promoting secondary wall formation after the establishment of tissue identity. Plant J 56: 768–778. 18657234 10.1111/j.1365-313X.2008.03633.x

[18] Wu A, Allu AD, Garapati P, Siddiqui H, Dortay H, et al (2012) JUNGBRUNNEN1, a reactive oxygen species-responsive NAC transcription factor, regulates longevity in *Arabidopsis.* . Plant Cell 24: 482–506. 22345491 10.1105/tpc.111.090894 PMC3315228

[19] Yang S, Seo P, Yoon H, Park C (2011) The *Arabidopsis* NAC transcription factor VNI2 integrates abscisic acid signals into leaf senescence via the *COR/RD* genes. Plant Cell 23: 2155–2168. 21673078 10.1105/tpc.111.084913 PMC3160032

[20] Uauy C, Distelfeld A, Fahima T, Blechl A, Dubcovsky J (2006) A NAC Gene regulating senescence improves grain protein, zinc, and iron content in wheat. Science 314: 1298–1301. 17124321 10.1126/science.1133649 PMC4737439

[21] Hao Y, Wei W, Song Q, Chen H, Zhang Y, et al (2011) Soybean NAC transcription factors promote abiotic stress tolerance and lateral root formation in transgenic plants. Plant J 68: 302–313. 21707801 10.1111/j.1365-313X.2011.04687.x

[22] Ozhuner E, Eldem V, Ipek A, Okay S, Sakcali S, et al (2013) Boron stress responsive microRNAs and their targets in barley. PLoS One 8: e59543. 23555702 10.1371/journal.pone.0059543 PMC3608689

[23] Nakashima K, Takasaki H, Mizoi J, Shinozaki K, Yamaguchi-Shinozaki K (2012) NAC transcription factors in plant abiotic stress responses. Biochim Biophys Acta 1819: 97–103. 22037288 10.1016/j.bbagrm.2011.10.005

[24] Tran L, Nishiyama R, Yamaguchi-Shinozaki K, Shinozaki K (2010) Potential utilization of NAC transcription factors to enhance abiotic stress tolerance in plants by biotechnological approach. GM Crops 1: 32–39. 21912210 10.4161/gmcr.1.1.10569

[25] Puranik S, Sahu PP, Srivastava PS, Prasad M (2012) NAC proteins: regulation and role in stress tolerance. Trends Plant Sci 17: 369–381. 22445067 10.1016/j.tplants.2012.02.004

[26] Tran LS, Nakashima K, Sakuma Y, Simpson SD, Fujita Y, et al (2004) Isolation and functional analysis of *Arabidopsis* stress-inducible NAC transcription factors that bind to a drought-responsive *cis*-element in the early responsive to dehydration stress 1 promoter. Plant Cell 16: 2481–2498. 15319476 10.1105/tpc.104.022699 PMC520947

[27] He X, Mu R, Cao W, Zhang Z, Zhang J, et al (2005) AtNAC2, a transcription factor downstream of ethylene and auxin signaling pathways, is involved in salt stress response and lateral root development. Plant J 44: 903–916. 16359384 10.1111/j.1365-313X.2005.02575.x

[28] Jensen MK, Rung JH, Gregersen PL, Gjetting T, Fuglsang AT, et al (2007) The HvNAC6 transcription factor: a positive regulator of penetration resistance in barley and *Arabidopsis.* . Plant Mol Biol 65: 137–150. 17619150 10.1007/s11103-007-9204-5

[29] Delessert C, Kazan K, Wilson IW, Van Der Straeten D, Manners J, et al (2005) The transcription factor ATAF2 represses the expression of pathogenesis-related genes in *Arabidopsis.* . Plant J 43: 745–757. 16115070 10.1111/j.1365-313X.2005.02488.x

[30] Lu P, Chen N, An R, Su Z, Qi B, et al (2007) A novel drought-inducible gene, ATAF1, encodes a NAC family protein that negatively regulates the expression of stress-responsive genes in *Arabidopsis.* . Plant Mol Biol 63: 289–305. 17031511 10.1007/s11103-006-9089-8

[31] Christianson JA, Wilson IW, Llewellyn DJ, Dennis ES (2009) The low-oxygen-induced NAC domain transcription factor ANAC102 affects viability of *Arabidopsis* seeds following low-oxygen treatment. Plant Physiol 149: 1724–1738. 19176720 10.1104/pp.108.131912 PMC2663757

[32] Hu H, You J, Fang Y, Zhu X, Qi Z, et al (2008) Characterization of transcription factor gene *SNAC2* conferring cold and salt tolerance in rice. Plant Mol Biol 67: 169–181. 18273684 10.1007/s11103-008-9309-5

[33] Nakashima K, Tran LS, Van Nguyen D, Fujita M, Maruyama K, et al (2007) Functional analysis of a NAC-type transcription factor OsNAC6 involved in abiotic and biotic stress-responsive gene expression in rice. Plant J 51: 617–630. 17587305 10.1111/j.1365-313X.2007.03168.x

[34] Zheng X, Chen B, Lu G, Han B (2009) Overexpression of a NAC transcription factor enhances rice drought and salt tolerance. Biochem Biophys Res Commun 379: 985–989. 19135985 10.1016/j.bbrc.2008.12.163

[35] Yokotani N, Ichikawa T, Kondou Y, Matsui M, Hirochika H, et al (2009) Tolerance to various environmental stresses conferred by the salt-responsive rice gene ONAC063 in transgenic *Arabidopsis.* . Planta 229: 1065–1075. 19225807 10.1007/s00425-009-0895-5

[36] Takasaki H, Maruyama K, Kidokoro S, Ito Y, Fujita Y, et al (2010) The abiotic stress-responsive NAC-type transcription factor OsNAC5 regulates stress-inducible genes and stress tolerance in rice. Mol Genet Genomics 284: 173–183. 20632034 10.1007/s00438-010-0557-0

[37] Hu H, Dai M, Yao J, Xiao B, Li X, et al (2006) Overexpressing a NAM, ATAF, and CUC (NAC) transcription factor enhances drought resistance and salt tolerance in rice. Proc Natl Acad Sci U S A 103: 12987–12992. 16924117 10.1073/pnas.0604882103 PMC1559740

[38] Gao F, Xiong A, Peng R, Jin X, Xu J, et al (2009) OsNAC52, a rice NAC transcription factor, potentially responds to ABA and confers drought tolerance in transgenic plants. Plant Cell Tiss Org 100: 255–262.

[39] Meng C, Cai C, Zhang T, Guo W (2009) Characterization of six novel NAC genes and their responses to abiotic stresses in *Gossypium hirsutum* L. Plant Sci 176: 352–359.

[40] Xie Q, Sanz-Burgos AP, Guo H, García JA, Gutiérrez C (1999) GRAB proteins, novel members of the NAC domain family, isolated by their interaction with a geminivirus protein. Plant Mol Biol 39: 647–656. 10350080 10.1023/a:1006138221874

[41] Xia N, Zhang G, Sun Y, Zhu L, Xu L, et al (2010) TaNAC8, a novel NAC transcription factor gene in wheat, responds to stripe rust pathogen infection and abiotic stresses. Physiol Mol Plant Pathol 74: 394–402.

[42] Xia N, Zhang G, Liu X, Deng L, Cai G, et al (2010) Characterization of a novel wheat NAC transcription factor gene involved in defense response against stripe rust pathogen infection and abiotic stresses. Mol Biol Rep 37: 3703–3712. 20213512 10.1007/s11033-010-0023-4

[43] Baloglu MC, Öz MT, Öktem HA, Yücel M (2012) Expression analysis of TaNAC69-1 and TtNAMB-2, wheat NAC family transcription factor genes under abiotic stress conditions in durum wheat (*Triticum turgidum*). Plant Mol Biol Rep 30: 1246–1252.

[44] Xue G, Neil IB, McIntyre CL, Riding GA, Kemal Kazan K, et al (2006) TaNAC69 from the NAC superfamily of transcription factors is up-regulated by abiotic stresses in wheat and recognises two consensus DNA-binding sequences. Funct Plant Biol 33: 43–57. 10.1071/FP05161 32689213

[45] Xue G, Way H, Richardson T, Drenth J, Joyce P, et al (2011) Overexpression of *TaNAC69* leads to enhanced transcript levels of stress up-regulated genes and dehydration tolerance in bread wheat. Mol Plant 4: 697–712. 21459832 10.1093/mp/ssr013

[46] Tang Y, Liu M, Gao S, Zhang Z, Zhao X, et al (2012) Molecular characterization of novel TaNAC genes in wheat and overexpression of *TaNAC2a* confers drought tolerance in tobacco. Physiol Plant 144: 210–24. 22082019 10.1111/j.1399-3054.2011.01539.x

[47] Mao X, Zhang H, Tian S, Chang X, Jing R (2010) TaSnRK2.4, an SNF1-type serine/threonine protein kinase of wheat (*Triticum aestivum* L.), confers enhanced multistress tolerance in *Arabidopsis.* . J Exp Bot 61: 683–96. 20022921 10.1093/jxb/erp331 PMC2814103

[48] Pang X, Mao X, Jing R, Shi J, Gao T, et al (2007) Analysis of gene expression profile in response to water stress in wheat (*Triticum aestivum* L.) seedlings. Acta Agron Sin 33: 333–336.

[49] Mao X, Kong X, Zhao G, Jia J (2005) Construction of a full-length cDNA library of *Aegilops speltoides* Tausch with optimized cap-trapper method. Acta genetica Sinica 32: 811–817. 16231735

[50] Jia J, Zhao S, Kong X, Li Y, Zhao G, et al (2013) *Aegilops tauschii* draft genome sequence reveals a gene repertoire for wheat adaptation. Nature 496: 91–95. 23535592 10.1038/nature12028

[51] Lescot M, Dehais P, Thijs G, Marchal K, Moreau Y, et al (2002) PlantCARE, a database of plant *cis-*acting regulatory elements and a portal to tools for *in silico* analysis of promoter sequences. Nucleic Acids Res 30: 325–327. 11752327 10.1093/nar/30.1.325 PMC99092

[52] Livaka KJ, Schmittgen TD (2001) Analysis of relative gene expression data using real-time quantitative PCR and the 2(-Delta Delta C(T)) method. Methods 25: 402–408. 11846609 10.1006/meth.2001.1262

[53] Ding Z, Li S, An X, Liu X, Qin H, et al (2009) Transgenic expression of *MYB15* confers enhanced sensitivity to abscisic acid and improved drought tolerance in *Arabidopsis thaliana.* . J Genet Genomics 36: 17–29. 19161942 10.1016/S1673-8527(09)60003-5

[54] Wang C, Jing R, Mao X, Chang X, Li A (2011) TaABC1, a member of the activity of bc1 complex protein kinase family from common wheat, confers enhanced tolerance to abiotic stresses in *Arabidopsis.* . J Exp Bot 62: 1299–12311. 21115661 10.1093/jxb/erq377 PMC3022413

[55] Hajdukiewicz P, Svab Z, Maliga P (1994) The small, versatile pPZP family of Agrobacterium binary vectors for plant transformation. Plant Mol Biol 25: 989–994. 7919218 10.1007/BF00014672

[56] Bent A (2006) *Arabidopsis thaliana* floral dip transformation method. Methods Mol Biol 343: 87–103. 16988336 10.1385/1-59745-130-4:87

[57] Hu C, Delauney AJ, Verma DP (1992) A bifunctional enzyme (delta 1-pyrroline-5-carboxylate synthetase) catalyzes the first two steps in proline biosynthesis in plants. Proc Natl Acad Sci U S A 89: 9354–9358. 1384052 10.1073/pnas.89.19.9354 PMC50125

[58] Fujita M, Fujita Y, Maruyama K, Seki M, Hiratsu K, et al (2004) A dehydration-induced NAC protein, RD26, is involved in a novel ABA-dependent stress-signaling pathway. Plant J 39: 863–876. 15341629 10.1111/j.1365-313X.2004.02171.x

[59] Strasser RJ, Sivastava A, Tsimilli-Michae ML (2002) The fluorescence transient as a tool to characterize and screen photosynthetic samples. In: Mohanty P, Yunus U and Pathre M, Editors, Probing Photosynthesis: Mechanism, regulation and adaptation, Taylor and Francis, London 443–480.

[60] Krause GH, Weis E (1991) Chlorophyll fluorescence and photosynthesis: the basics. Annu Rev Plant Physiol Plant Mol Biol 42: 313–349.

[61] Levitt J (1980) Responses of plants to environmental stresses. In: Water, radiation, salt and other stresses, vol. II, Academic Press, New York 3–211.

[62] Farooq S, Azam F (2006) The use of cell membrane stability (CMS) technique to screen for salt tolerant wheat varieties. J Plant Physiol 163: 629–637. 16545996 10.1016/j.jplph.2005.06.006

[63] Shabala S (2006) Non-invasive microelectrode ion flux measurements in plant stress physiology. In: Volkov A (ed) Plant Electrophysiology - Theory and methods. Springer-Verlag, Berlin 35–71.

[64] Wakeel A, Sumer A, Hanstein S, Yan F, Schubert S (2011) *In vitro* effect of different Na^+^/K^+^ ratios on plasma membrane H^+^-ATPase activity in maize and sugar beet shoot. Plant Physiol Biochem 49: 341–345. 21282062 10.1016/j.plaphy.2011.01.006

[65] Chen Z, Pottosin II, Cuin TA, Fuglsang AT, Tester M, et al (2007) Root plasma membrane transporters controlling K^+^/Na^+^ homeostasis in salt-stressed barley. Plant Physiol 145: 1714–1725. 17965172 10.1104/pp.107.110262 PMC2151677

[66] Cuin TA, Zhou M, Parsons D, Shabala S (2011) Genetic behaviour of physiological traits conferring cytosolic K^+^/Na^+^ homeostasis in wheat. Plant Biol (Stuttg) 14: 438–446. 22117736 10.1111/j.1438-8677.2011.00526.x

[67] Chen Z, Newman I, Zhou M, Mendham N, Zhang G, et al (2005) Screening plants for salt tolerance by measuring K^+^ flux: a case study for barley. Plant Cell Environ 28: 1230–1246.

[68] Chen Z, Zhou M, Newman IA, Mendham NJ, Zhang G, et al (2007) Potassium and sodium relations in salinised barley tissues as a basis of differential salt tolerance. Funct Plant Biol 34: 150–162. 10.1071/FP06237 32689341

[69] Cuin TA, Betts SA, Chalmandrier R, Shabala S (2008) A root's ability to retain K^+^ correlates with salt tolerance in wheat. J Exp Bot 59: 2697–2706. 18495637 10.1093/jxb/ern128 PMC2486465

[70] Hao Y, Song Q, Chen H, Zou H, Wei W, et al (2010) Plant NAC-type transcription factor proteins contain a NARD domain for repression of transcriptional activation. Planta 232: 1033–1043. 20683728 10.1007/s00425-010-1238-2

[71] Liu Q, Kasuga M, Sakuma Y, Abe H, Miura S, et al (1998) Two transcription factors, DREB1 and DREB2, with an EREBP/AP2 DNA binding domain separate two cellular signal transduction pathways in drought- and low-temperature-responsive gene expression, respectively, in *Arabidopsis.* . Plant Cell 10: 1391–406. 9707537 10.1105/tpc.10.8.1391 PMC144379

[72] Xiong L, Ishitani M, Zhu J (1999) Interaction of osmotic stress, temperature, and abscisic acid in the regulation of gene expression in *Arabidopsis.* . Plant Physiol 119: 205–212. 9880362 10.1104/pp.119.1.205 PMC32221

[73] Finkelstein RR, Somerville CR (1990) Three classes of abscisic acid (ABA)-insensitive mutations of *Arabidopsis* define genes that control overlapping subsets of ABA responses. Plant Physiol 94: 1172–1179. 16667813 10.1104/pp.94.3.1172 PMC1077358

[74] Medina J, Bargues M, Terol J, Perez-Alonso M, Salinas J (1999) The *Arabidopsis CBF* gene family is composed of three genes encoding AP2 domain-containing proteins whose expression Is regulated by low temperature but not by abscisic acid or dehydration. Plant Physiol 119: 463–470. 9952441 10.1104/pp.119.2.463 PMC32122

[75] Dubouzet JG, Sakuma Y, Ito Y, Kasuga M, Dubouzet EG, et al (2003) *OsDREB* genes in rice, *Oryza sativa* L., encode transcription activators that function in drought-, high-salt- and cold-responsive gene expression. Plant J 33: 751–763. 12609047 10.1046/j.1365-313x.2003.01661.x

[76] Yamaguchi-Shinozaki K, Shinozaki K (1993) Characterization of the expression of a desiccation-responsive *rd29* gene of *Arabidopsis thaliana* and analysis of its promoter in transgenic plants. Mol Gen Genet 236: 331–340. 8437577 10.1007/BF00277130

[77] Lang V, Palva ET (1992) The expression of a rab-related gene, *rab18*, is induced by abscisic acid during the cold acclimation process of *Arabidopsis thaliana* (L.) Heynh. Plant Mol Biol 20: 951–962. 1463831 10.1007/BF00027165

