# Supplementary material for: Novel NAC Transcription Factor TaNAC67 Confers Enhanced Multi-Abiotic Stress Tolerances in Arabidopsis
Source: PLoS One. 2014 Jan 10;9(1):e84359. doi: 10.1371/journal.pone.0084359 (PMC3888409; doi:10.1371/journal.pone.0084359)
Supplement: File S1 — Fig. S1, Expression levels of TaNAC67 in different transgenic Arabidopsis lines. Gene expression levels of TaNAC67 differed significantly in different transgenic Arabidopsis lines. L1 - 6, TaNAC67 transgenic lines. The expression of TaNAC67 in L1 was regarded as a standard for its lower expression level. Fig. S2, Comparison of primary root lengths and lateral root numbers for TaNAC67 transgenics and the two controls. A. Phenotype of primary roots for TaNAC67 transgenics. B. The primary root lengths of most transgenic plants were significantly shorter than the WT and GFP controls. C. Lateral root numbers of trangenics and controls were not significantly different. Arabidopsis plants were sown on MS plates solidified with 1.0% agar and cultured vertically in a greenhouse. Primary root length and lateral root numbers were measured after 10 d and 14 d, respectively. *, significantly different at P = 0.05. Values are means ± SE (n = 20). Fig. S3, No differences were identified in biomass of TaNAC67 transgenics and two controls. Arabidopsis plants were grown in sieve-like rectangular containers filled with mixed soil (vermiculite: humus = 1∶1) and cultured under well-watered conditions as described in Materials and Methods. Six plants were collected per sample for biomass measurement; four replications were set for each Arabidopsis line and the plants were harvested at five weeks. Single plant biomasses were calculated before and after treatment in an 80°C oven for 24 h. FW, fresh weight; DW, dry weight. Four replications were performed, and values are means ± SE. Fig. S4, A rapid decrease, slow increase and slow decrease pattern in chlorophyll content was identified Arabidopsis after exposure to high salinity. The chlorophyll contents were measured at designated times after exposure to 300 mM NaCl solution. Twenty plants were measured for each line. Values are means ± SE (n = 20). Fig. S5, No differences were identified in free proline contents for TaNAC67 transgenics an [file pone.0084359.s001.docx]

**Novel NAC transcription factor TaNAC67 confers enhanced multi-stress tolerances in *Arabidopsis***

Xinguo Mao, Shuangshuang Chen, Ang Li, Chaochao Zhai, Ruilian Jing^*^

The Key Laboratory for Crop Gene Resources and Germplasm Enhancement, Ministry of Agriculture; The National Key Facility for Crop Gene Resources and Genetic Improvement; Institute of Crop Science, Chinese Academy of Agricultural Sciences, Beijing 100081, China

*Corresponding author

Contact Information

Ruilian Jing

Institute of Crop Science

Chinese Academy of Agricultural Sciences

Beijing 100081

China

Tel/Fax: 86-10-82105829

Email: [jingrl@caas.net. cn](mailto:jingrl@caas.net.%20cn)

**Supplementary information**


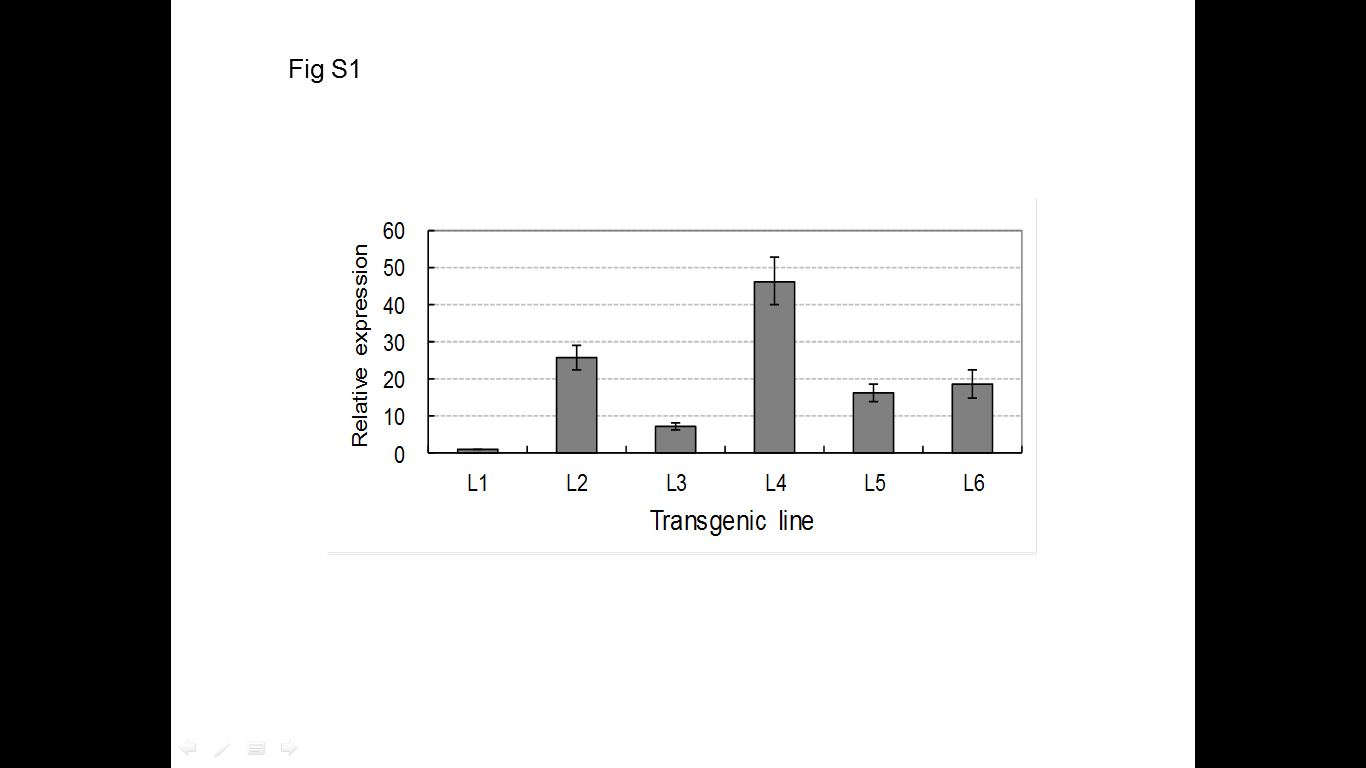


**Fig. S1 Expression levels of *TaNAC67* in different transgenic *Arabidopsis* lines**

Gene expression levels of *TaNAC67* differed significantly in different transgenic *Arabidopsis* lines. L1 - 6, *TaNAC67* transgenic lines. The expression of *TaNAC67* in L1 was regarded as a standard for its lower expression level.


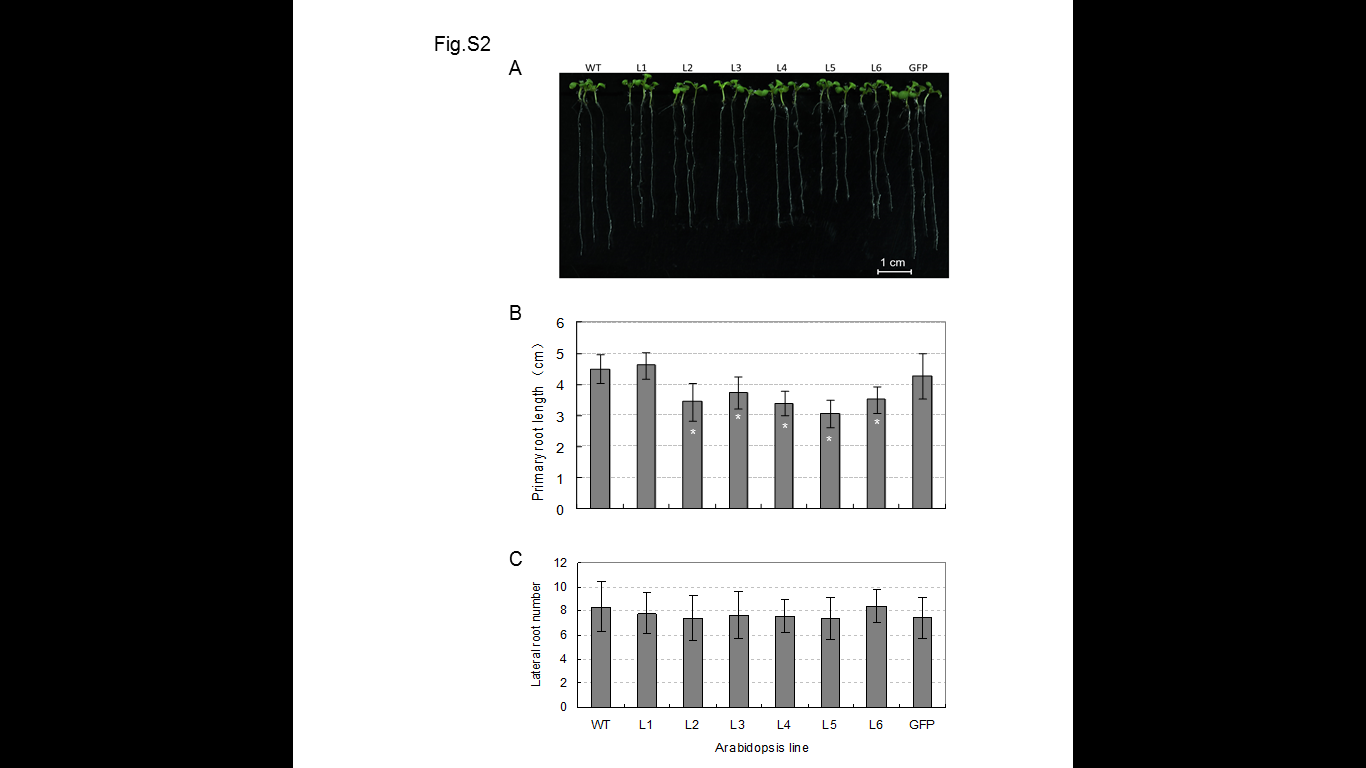


**Fig. S2 Comparison of primary root lengths and lateral root numbers for *TaNAC67* transgenics and two controls**

A. Phenotype of primary roots for *TaNAC67* transgenics. B. The primary root lengths of most transgenic plants were significantly shorter than the WT and GFP controls. C. Lateral root numbers of trangenics and controls were not significantly different.

*Arabidopsis* plants were sown on MS plates solidified with 1.0% agar and cultured vertically in a greenhouse. Primary root length and lateral root numbers were measured after 10 d and 14 d, respectively. *, significantly different at *P*=0.05. Values are means ± SE (n=20).

**Fig. S3 No differences were identified in biomass of *TaNAC67* transgenics and the two controls**

*Arabidopsis* plants were grown in sieve-like rectangular containers filled with mixed soil (vermiculite : humus = 1:1) and cultured under well-watered conditions as described in Materials and Methods. Six plants were collected per sample for biomass measurement; four replications were set for each *Arabidopsis* line and the plants were harvested at five weeks. Single plant biomasses were calculated before and after treatment in an 80 ℃ oven for 24 h. FW, fresh weight; DW, dry weight. Four replications were performed, and values are means ± SE.


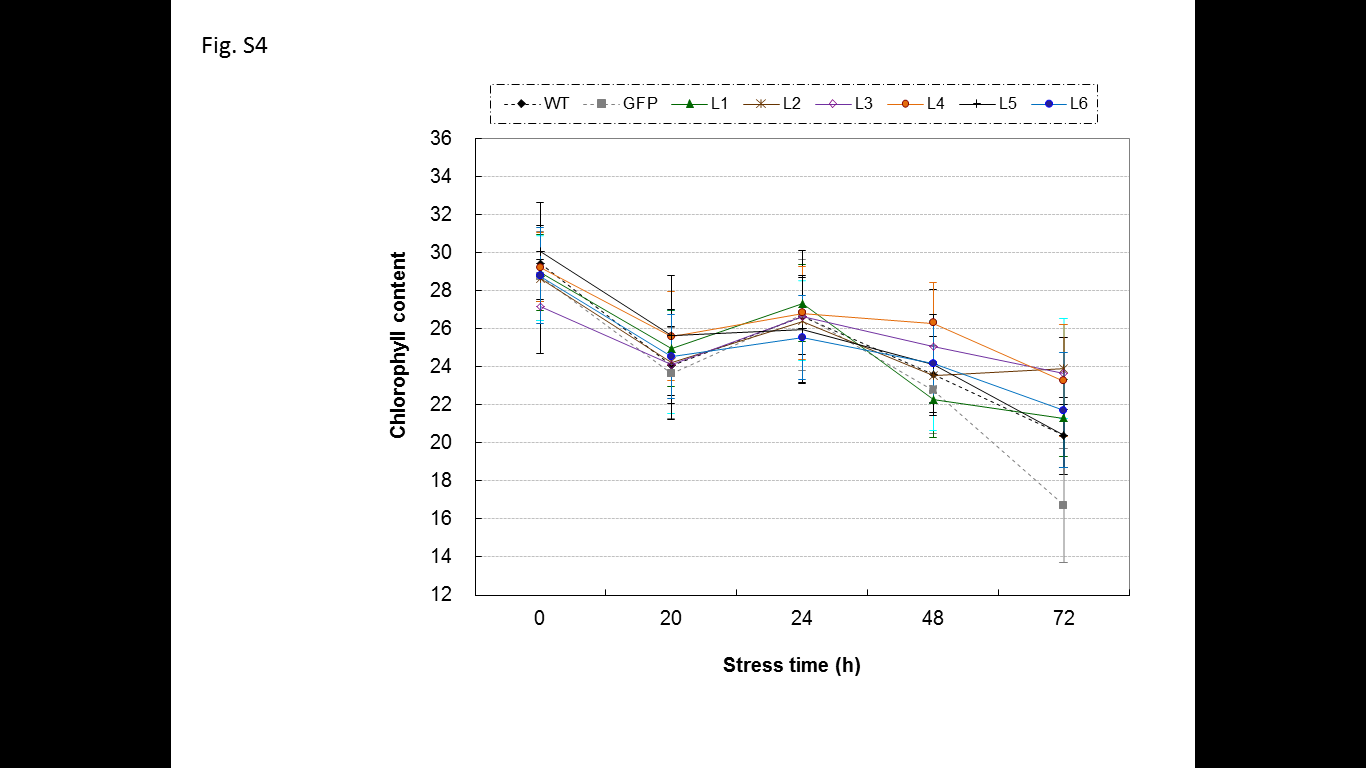


**Fig. S4 A rapid decrease, slow increase and slow decrease pattern in chlorophyll content was identified Arabidopsis after exposure to high salinity.**

The chlorophyll contents were measured at designated times after exposure to 300 mM NaCl solution. Twenty plants were measured for each line. Values are means ± SE (n=20).


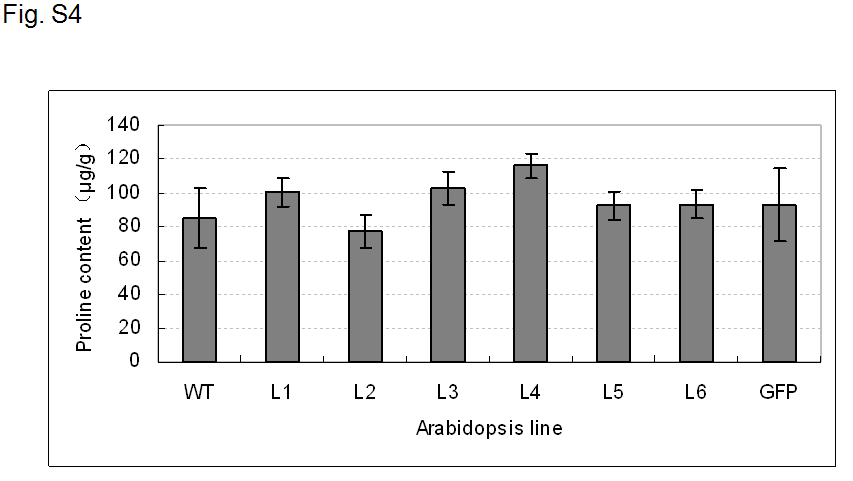


**Fig. S5 No differences were identified in free proline contents for *TaNAC67* transgenics and the two controls**

*Arabidopsis* plants were cultured as described in Materials and Methods. Five plants were collected as a single sample for measurement of free proline content. The experiment consisted of three replications. Values are means ± SE.

**Table S1 Plant materials used for identification of genomic origins**

Accession numbers are for the Chinese National Gene Bank, CAAS.

| **Species** | **Accession no. or variety** | **Genome** | **Origin** | **Species** | **Accession no. or variety** | **Genome** | **Origin** |
| --- | --- | --- | --- | --- | --- | --- | --- |
| *T*. *urartu* | UR 201 | AA | Lebanon | *Ae*. *tauschii* | Y 92 | DD | China |
| *T*. *urartu* | UR 204 | AA | Lebanon | *Ae*. *tauschii* | Y 215 | DD | Mexico |
| *T*. *urartu* | UR 206 | AA | Lebanon | *T. dicoccoides* | DS1 | AABB | France |
| *T*. *urartu* | UR 209 | AA | Syria | *T. dicoccoides* | DS6 | AABB | Germany |
| *Ae. speltoides* | Y 2003 | SS | Syria | *T. dicoccoides* | DS10 | AABB | Canada |
| *Ae. speltoides* | Y 2009 | SS | Syria | *T. dicoccum* | DM 51 | AABB | Canada |
| *Ae. speltoides* | Y 2017 | SS | Syria | *T. aestivum* | Hanxuan 10 | AABBDD | China |
| *Ae. speltoides* | Y 2021 | SS | Iran | *T. aestivum* | Chinese Spring | AABBDD | China |
| *Ae*. *tauschii* | AE 38 | DD | Iran | *T. aestivum* | Opata 85 | AABBDD | Mexico |
| *Ae*. *tauschii* | AE 46 | DD | China | *T. aestivum* | W7984 | AABBDD | Mexico |

**Table S2. Primer pairs used in quantitative real-time PCR in *Arabidopsis***

| **Gene** | **Accession no.** | **Forward primer** | **Reverse primer** |
| --- | --- | --- | --- |
| *ABA1* | AT5G67030 | GCTATGAAGGTGATCTGCTTGTGG | TTCATACCATTTGGAGCATCAGC |
| *ABI1* | AT4G26080 | AGCTGCTGATATAGTCGTCGTTGATA | GAGGATCAAACCGACCATCTAACA |
| *ABI2* | AT5G57050 | GTTCTTGTTCTGGCGACGGAGC | CCATTAGTGACTCGACCATCAAG |
| *ABI5* | AT2G36270 | AGAGGGATAGCGAACGAGTCTAGTC | GTTCGGGTTTGGATTAGGTTTAGG |
| *CBF1* | AT4G25490 | GTTTGGGATGCCGACTTTGTT | ACCATCTCCTTCGCCGTCAT |
| *CBF2* | AT4G25470 | GGGACTTTCCAAACCGCTGAG | GGATTTCCTTGGCACAGGTTGA |
| *COR15A* | AT2G42540 | GTGACGGATAAAACAAAAGAGG | GACCCTACTTTGTGGCATCCTT |
| *COR47* | AT1G20440 | ATGGCTGAGGAGTACAAGAACAACGTT | TCTTCTTCTTCTTCTCCTTCTTTTCCT |
| *DREB1A* | AT4G25480 | TGCGTTGGCGTTTCAGGATG | CAAACTCGGCATCTCAAACATCG |
| *DREB2A* | AT5G05410 | CTGGAGAATGGTGCGGAAGA | CAGATAGCGAATCCTGCTGTTGT |
| *RAB18* | AT5G66400 | GCAGTATGACGAGTACGGAAATCC | CCTTGTCCATCATCCGAGCTAGA |
| *RD22* | AT5G25610 | ACTTGGTAAATATCACGTCAGGGCT | CTGAGGTGTTCTTGTGGCATACC |
| *RD29A* | AT5G52310 | GATAACGTTGGAGGAAGAGTCGG | TCCTGATTCACCTGGAAATTTCG |
| *RD29B* | AT5G52300 | CCGACAAGAGGTGATGTGAAAGTAG | GTGTAACCTAGCTTTGAGGCAACG |
| *Actin* | AT3G18780 | TATCGCTGACCGTATGAGCAAAG | TGGACCTGCCTCATCATACTCG |
